# Kinetic comparison of all eleven viral polyprotein cleavage site processing events by SARS-CoV-2 main protease using a linked protein FRET platform

**DOI:** 10.1016/j.jbc.2024.107367

**Published:** 2024-05-15

**Authors:** Calem Kenward, Marija Vuckovic, Mark Paetzel, Natalie C.J. Strynadka

**Affiliations:** 1 Department of Biochemistry and Molecular Biology and Centre for Blood Research, The University of British Columbia, Vancouver, British Columbia, Canada; 2 Department of Molecular Biology and Biochemistry, Simon Fraser University, Burnaby, British Columbia, Canada

**Keywords:** SARS-CoV-2, FRET, viral protease, protease substrate, main protease, Mpro, 3CL protease, 3CLpro, polyprotein, enzyme kinetics, coronavirus

## Abstract

The main protease (M^pro^) remains an essential therapeutic target for COVID-19 post infection intervention given its critical role in processing the majority of viral proteins encoded by the genome of severe acute respiratory syndrome related coronavirus 2 (SARS-CoV-2). Upon viral entry, the +ssRNA genome is translated into two long polyproteins (pp1a or the frameshift-dependent pp1ab) containing all the nonstructural proteins (nsps) required by the virus for immune modulation, replication, and ultimately, virion assembly. Included among these nsps is the cysteine protease M^pro^ (nsp5) which self-excises from the polyprotein, dimerizes, then sequentially cleaves 11 of the 15 cut-site junctions found between each nsp within the polyprotein. Many structures of M^pro^ (often bound to various small molecule inhibitors or peptides) have been detailed recently, including structures of M^pro^ bound to each of the polyprotein cleavage sequences, showing that M^pro^ can accommodate a wide range of targets within its active site. However, to date, kinetic characterization of the interaction of M^pro^ with each of its native cleavage sequences remains incomplete. Here, we present a robust and cost-effective FRET based system that benefits from a more consistent presentation of the substrate that is also closer in organization to the native polyprotein environment compared to previously reported FRET systems that use chemically modified peptides. Using this system, we were able to show that while each site maintains a similar Michaelis constant, the catalytic efficiency of M^pro^ varies greatly between cut-site sequences, suggesting a clear preference for the order of nsp processing.

The 30kb (+)ssRNA genome of severe acute respiratory syndrome related coronavirus-2 (SARS-CoV-2) inherently encodes two polyproteins which must undergo intramolecular processing by two integral viral proteases, main protease (M^pro^) and papain like protease (PL^pro^), that specifically cleave the polyproteins at multiple sites (Fig. 1*A*) (1, 2). The two polyproteins produced, pp1a (490 kDa) and the longer pp1ab (794 kDa—the product of a ribosomal frameshift) (3, 4), encompass all of the nonstructural proteins (nsp) required for host manipulation, replication, and maintenance of the viral lifecycle. Processing of viral polyproteins is thought not to occur randomly across the different cleavage sites as the coordination of polyprotein processing by viral proteases is a key regulatory event in the life cycle of most +ssRNA viruses (5, 6), including SARS-CoV-2 (7), with coordinated processing of pp1a shown to be crucial for replication (8). This tight regulatory control is part of a larger set of mechanisms that underly viral replication and proliferation used by most RNA viruses and retroviruses with polyprotein precursors (9, 10). The overlapping gene organization encoding for these polyproteins allows for a more compact genome and regulation of activity through both precise temporal (*i.e.*, stage of viral cycle) and spatial (*i.e.*, subcellular location) control. This allows for the release of protein subsets with different biochemical functions from the same precursor ensues, as previously observed for related alphaviruses, picornaviruses, and noroviruses (5, 11). Intermediates from polyprotein processing have previously been observed during murine hepatitis virus (12, 13) and alphacoronavirus human CoV 229E (HCoV-229E) infections (14). Stepwise cleavage of the viral polyprotein has also been characterized in SARS-CoV-1 (15) and SARS-CoV-2 (10), further highlighting the importance of coordinated polyprotein processing in the viral lifecycle.

**Figure 1:**
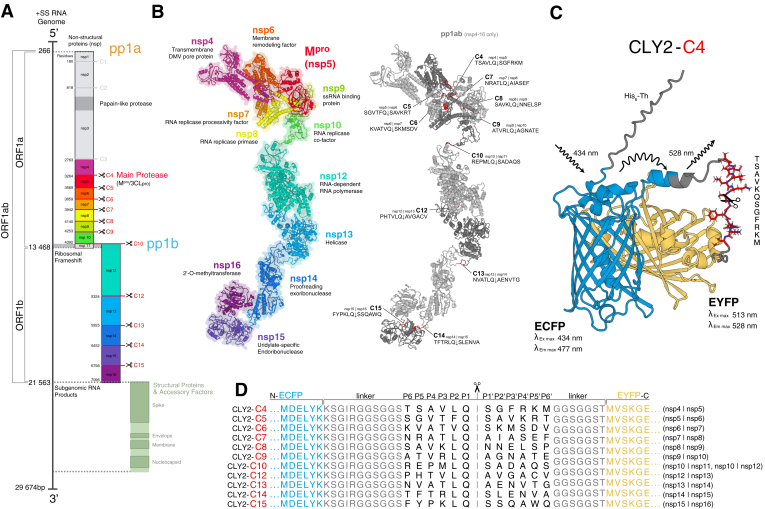
**Organization of SARS-CoV-2 polyprotein cleavage sites dictates design of FRET reporter substrates.***A*, schematic overview of the SARS-CoV-2 +ssRNA genome. Open reading frames (ORF) 1a and 1b encode polyproteins pp1a and pp1ab which must be processed by two self-encoded viral proteases, PLpro and M^pro^ (nsp3/nsp5 respectively). Of the 15 polyprotein cleavage sites, M^pro^ is responsible for processing 11 (C4-10 and C12-C15, highlighted in *red*). *B*, Alphafold2 (106, 107) generated monomer model of pp1ab polyprotein processed by M^pro^ (nsp4-nsp16) with cleavage sequences annotated. *C*, predicted model of ECFP-EYFP FRET pair joined with C4 (nsp4/nsp5 junction) M^pro^ cleavage site sequence. Hexahistidine and thrombin protease cleavage site expression tag (His_6_-Th) shown attached to C terminus of ECFP. Excitation of ECFP at 434 nm stimulates fluorescence of EYFP at 528 nm. Cleavage of C4 sequence disconnects ECFP from EYFP resulting in measurable decrease in EYFP fluorescence and proportional increase in ECFP fluorescence. *D*, sequence alignment of the SARS-CoV-2 M^pro^ cleavage-site specificity residues (P6–P6′) inserted into linker of ECFP-Cx-EYFP FRET system, shown in *light gray*. CLYx, ECFP- linker-EYFP protein with a linker containing x GGSGGS repeats; ECFP, enhanced cyan fluorescent protein; EYFP, enhanced yellow fluorescent protein; PLpro, papain like protease; SARS-CoV-2, severe acute respiratory syndromerelated coronavirus 2.

In SARS-CoV-1/2, following translation by host machinery, pp1a/pp1ab is consequently processed into 16 smaller nsps (nsp1 to nsp16), by the two self-encoded cysteine proteases PL^pro^ (nsp3) and M^pro^ (also called 3C-like protease/nsp5) (16, 17). M^pro^ is responsible for the majority of these processing events, cleaving 11 of the 16 highly conserved recognition sites (17) on the replicase polyproteins found between nsp4 and nsp16 (Figs. 1 and S1). After self-excision from the polyprotein and dimerization (18, 19, 20), M^pro^ subsequently liberates nsps 6 to 16 from the polyprotein by specifically targeting a conserved motif in nsp interdomain junctions. These polyprotein cleavage sites primarily consist of a consensus sequence of Q↓ (S/A/G/N) at the P1↓P1' positions (where ↓ denotes the peptide bond cleavage location, following Schechter–Berger annotation nomenclature), but the only strictly conserved requirement is a glutamine at P1 which is invariant among different coronaviruses (see Table S1) (5, 6, 7, 14). Beyond this requirement, examination of SARS-CoV-1 and SARS-CoV-2 cut-site sequences indicates a preference for a hydrophobic residue at P2 (typically leucine), and restriction to a small, generally aliphatic P4, and either a serine, alanine, glycine, or asparagine in P1′ (21, 22). Between SARS-CoV-2 and SARS-CoV-1 and their associated variants, there is a little variation between the cut-site sequences. Expanding to other human pathogenic Coronaviridae, it is apparent that variability within these cleavage site regions is tolerated by M^pro^ but the conservation of motifs within certain junctions is suggestive of their contribution to the order of polyprotein processing. For example, C9 is almost entirely conserved, while C13 is highly variable with only the canonical glutamine remaining (Fig. S1 and Table S1). M^pro^ has also been shown to have a wide range of nonviral targets, further highlighting the promiscuity of M^pro^ which plays an important role in interference of key cellular host factors to enhance viral replication (23), modulation of the host immune response, and viral pathogenicity (24, 25).

Due to its essential role, high degree of conservation, and the absence of closely related homologs in the human genome, M^pro^ has emerged as an attractive target for the development of antiviral therapeutics and has been extensively investigated to determine the interplay between M^pro^ structure and proteolytic activity (26). Many inhibitors have been designed that specifically target M^pro^ which ultimately disrupt viral replication and reduce the severity of coronavirus disease 2019 (27, 28, 29, 30, 31). Initial candidate inhibitors were those previously developed for SARS-CoV-1 (32, 33, 34) other coronaviruses (28, 35), or other viral proteases (36, 37). This includes the most broadly used M^pro^ inhibitor to date, nirmatrelvir (PF-07321332), which is a reversible covalent inhibitor that utilizes a nitrile warhead to target the catalytic cysteine (32) and was derived from a potent inhibitor of M^pro^ from SARS-CoV-1 (38). Recently, structure based screening efforts have led to the development of new classes of novel inhibitors against SARS-CoV-2 M^pro^, work that exploited recent advances in understanding of both the binding landscape and kinetics of M^pro^ (30, 38, 39, 40). However, there is still a need for a comprehensive understanding of the kinetics which allow M^pro^ to effectively interact with such a wide breadth of cleavage targets, how differences in target cleavage sequences impact the catalytic efficiency, and how these interactions may govern pp1a/pp1ab processing, and by extension the lifecycle of SARS-CoV-2.

The rapid development of M^pro^ inhibitors has heavily relied on utilizing FRET based screening methods with peptide substrates to monitor protease activity (28, 29, 41, 42, 43). FRET systems have been extensively used for probing protein-protein interactions and studying enzyme kinetics as it allows for real-time monitoring of molecular interactions, conformational changes, and enzymatic activities with high sensitivity (44, 45). A number of M^pro^ FRET enzyme assays have been developed using different substrates, M^pro^ constructs, and buffer conditions (36, 41, 46, 47), but inconsistent methodologies has led to varied results (summarized in Table S2), including when screening potential M^pro^ inhibitors (41, 48, 49). Efforts to develop an improved SARS-CoV-2 M^pro^ assay that delivers improved consistency while maintaining high sensitivity are ongoing (41, 50); however, the use of peptidomimetic substrates linked with small molecular fluorophores (*e.g.* EDANS/Dabcyl (46), FAM/Dabcyl (50), MCA/Dnp (51), and so on.) remains problematic. As an alternative, fluorescent proteins connected *via* a flexible peptide linker are available as FRET sensors (52, 53). Other FRET systems have been developed that utilize a flexible polypeptide that undergoes conformational changes upon analyte binding (53, 54, 55) or incorporate linker peptides with protease-specific sequences (56, 57).

Here, we present a novel FRET-based system used to characterize the enzymatic activity, determine kinetic parameters, and gain insights into the catalytic mechanism of the main protease of SARS-CoV-2. To ensure a more consistent presentation of the substrate recognition sequence to the M^pro^ binding site, we have employed the use of a tethered peptide substrate design (Fig. 1*C*) (58), inserting a fixed range of twelve residues (P6-P6’) corresponding to each polyprotein cut-site (Cx) into a flexible linker in between two large fluorescent proteins, forming the ECFP-C*x-*EYFP FRET system (Fig. 1*D*). We propose that this tethered peptide approach more closely mimics the localization of cleavage site positions within the polyprotein, with the added benefit of providing economy and reproducibility *versus* synthesized peptide substrates. Upon addition of enzymatically active M^pro^, cleavage of the target sequence will separate the FRET pair, resulting in a measurable real-time change in fluorescence and therefore provide a sensitive measure of M^pro^ activity. Using this improved system, here, we provide the steady state kinetics of M^pro^ interacting with each native cut-site sequencing within the polyprotein, while also presenting a robust FRET-based system for characterizing interactions between M^pro^ and various host and other peptide targets. This method also allows for the investigation of the effects of mutations within M^pro^ from emerging variants. Together, these insights may contribute to the future design of more effective inhibitors and therapeutic strategies against SARS-CoV-2 and emerging variants.

## Results

### Design and production of ECFP-linker-EYFP M^pro^ substrates

A series of M^pro^ FRET substrates were prepared consisting of a fluorophore and quencher pair separated by one of the 11 SARS-CoV-2 polyprotein cleavage sequences targeted by M^pro^ (C4-C10, C12-C15; Fig. 1*D* see for definition of cleavage site nomenclature). The previously established ECFP- linker-EYFP protein with a linker containing x GGSGGS repeats system, comprised of an enhanced cyan fluorescent protein (ECFP) linked by a flexible peptide region of (GGSGGS)_n_ repeats to an enhanced yellow fluorescent protein (EYFP), was used as a template for our construct design due to its high efficiency as a FRET pair, ease of use, and ability to readily accommodate a range of residues within the linker region (58). Here, CLY2 contains 29 residues within the linker region: 13 residues are located between the last residue of ECFP and the first glycine residue of two GGSGGS repeats, with four additional residues located between the last serine residue of the tandem repeat and the first residue of EYFP (Fig. 1*C*).

Using the NcoI/EcoRI restriction sites included in the modified pET28-CLY2 plasmid, we cloned a series of fusion proteins by inserting twelve residues from each cut-site sequence (corresponding to M^pro^ cleavage-site specificity residues P6-P6’) in-between GGSGGS repeats of the CLY2 construct to form the ECFP-C*x*-EYFP system shown in Figure 1*D*. With the addition of the 12 cut-site residues, our constructs are equivalent to CLY4 in linker length, with a calculated radius of ∼45 Å separating ECFP and EYFP, resulting in a reported energy-transfer efficiency of 0.58 (58). Each of these substrate constructs was cloned into a modified pET28a plasmid including a N-terminal 6xHis-tag with a thrombin protease cleavage site to facilitate purification and subsequent removal of the expression tag. Each plasmid was recombinantly expressed in *Escherichia coli* and purified, resulting in multimilligram amounts (>50 mg per liter of culture) of each substrate construct which were an intense neon yellow in appearance throughout the entire purification process. Following lysis and affinity chromatography, each substrate was purified to isolation by size exclusion chromatography as confirmed by SDS-PAGE (Fig. S2). A subset of substrates was also further validated with mass spectrometry to confirm the molecular weight and presence of C4, C6, and C15 sequences (Fig. S3).

### Characterization of the ECFP-C*x*-EYFP M^pro^ substrates

Full-length SARS-CoV-2 M^pro^ enzyme with native N and C termini intact was produced recombinantly in BL21 *E.coli* using previously described methods as per prior structural and enzymatic studies (21, 27, 29, 30). To confirm the WT M^pro^ activity against these newly prepared fluorescent substrates, cleavage of each ECFP-C*x*-EYFP substrate by M^pro^ was monitored by SDS-PAGE, by mixing 25 μM of each substrate with 100 nM of M^pro^ and taking SDS-PAGE samples before (Fig. S4*A*) and after incubation overnight at room temperature (Fig. S4*B*). Interestingly, even in an end point condition, three of the substrates do not cleave completely, with C6, C8, and C10 each having a remaining upper band corresponding to <10% uncleaved substrate. All other substrates appear to cleave to completion. Cleavage of ECFP-C4-EYFP was interrogated in more detail with samples taken at regular intervals throughout the reaction to monitor the rate of substrate cleavage by M^pro^ (Fig. S5*A*). The resulting SDS-PAGE gel and densitometry analysis show a clear disappearance of the upper 60 kDa band and equivalent appearance of two smaller bands around 30 kDa corresponding to the generation of free ECFP and free EYFP (Fig. S5*B*).

**Figure 2:**
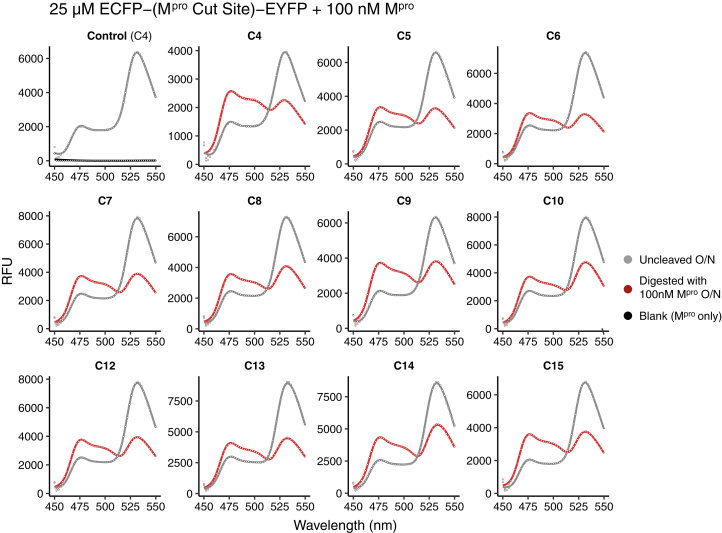
**Fluorescence emission wavelength scan shows cleavage of ECFP-C*x*-EYFP constructs by M**^**pro**^**.** Pairs of 10 μM samples of each ECFP-C*x*-EYFP construct were incubated overnight at room temperature, either with (*red*) or without (*gray*) 100 nM M^pro^ added. A clear shift from *gray* to *red* emission curves is evident in each case, with increase at 477 nm and proportional decrease in peak height at 528 nm as the EYFP-ECFP FRET pair is separated after the linker in cleaved by M^pro^. A control sample (*top left*) of CLY2 was examined using fresh ECFP-C4-EYFP to compare for any degradation of uncleaved samples overnight. Another sample of 100 nM M^pro^ without added substrate was also measured to verify the contribution of M^pro^ to the measured fluorescence (shown in *black* in control run; *top left*). ECFP, enhanced cyan fluorescent protein; EYFP, enhanced yellow fluorescent protein; M^pro^, main protease.

ECFP and EYFP form a fluorescent quenching pair and exhibit FRET within the construct when linked. With excitation at 434 nm, ECFP fluorescence at 477 nm is quenched and EYFP fluorescence at 528 nm is observed instead (as seen in an emission wave scan of ECFP-C4-EYFP in Fig. S5*C*). When the inserted substrate specificity sequence is cleaved by M^pro^, the FRET disappears and results in a decrease in EYFP fluorescence and a proportional increase in ECFP emission (Figs. 2 and S5, *C–F*). By monitoring these changes in fluorescence, the per substrate enzyme activity can be detected at subnanomolar protein concentration with sufficient sensitivity to characterize M^pro^ activity.

**Figure 3:**
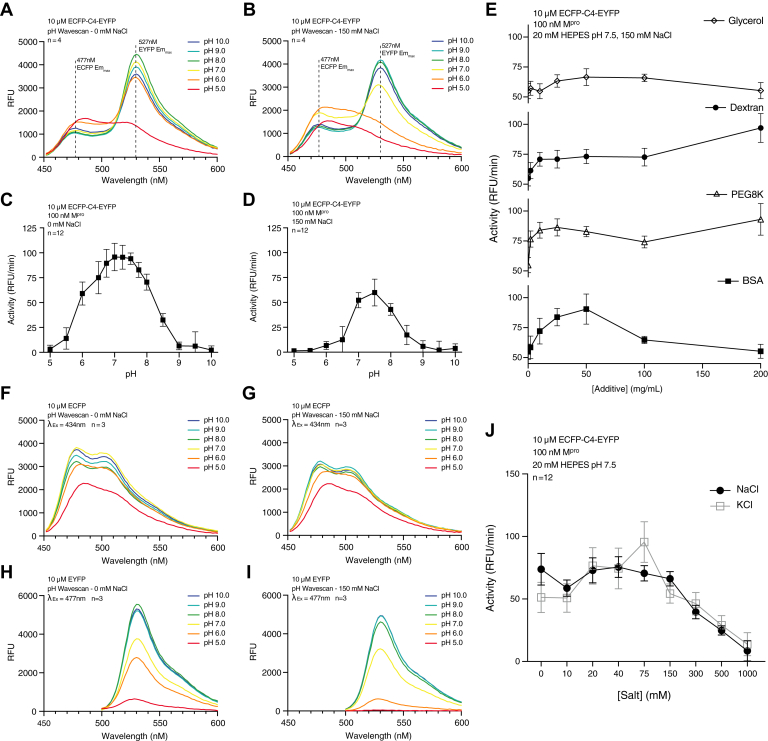
**Effect of buffer composition on M**^**pro**^**catalytic activity.***A* and *B*, emission scan from 450 nm to 600 nm of 10 μM ECFP-C4-EYFP after excitation at 434 nm with (*A*) 0 mM NaCl or (*B*) 150 mM NaCl included in buffer at pH 5 (*red*) to pH 10 (*blue*) indicated by color. All pH screening assays were performed with samples prepared in a multicomponent buffer of 20 mM Hepes, 20 mM Ches, and 20 mM citrate, adjusted to indicated pH with NaOH prior to final dilution. Rate of cleavage of 10 μM ECFP-C4-EYFP by 100 nM M^pro^ with (*C*) 0 mM or (*D*) 150 mM additional NaCl include din assay buffer. Each point represents mean of n = 12 and standard deviation shown as error bars. *E*, effect of various concentrations of molecular crowders on rate of cleavage of 10 μM ECFP-C4-EYFP by 100 mM M^pro^ in 20 mM Hepes, pH 7.5, 150 mM NaCl. *F* and *G*, emission scans from 450 nm to 600 nm of 10 μM free ECFP after excitation at 434 nm with (*F*) 0 mM NaCl or (G) 150 mM NaCl. included in buffer. *H* and *I*, emission scans from 500 nm to 600 nm of 10 μM free EYFP after excitation at 477 nm with (*H*) 0 mM NaCl or (*I*) 150 mM NaCl included in buffer. *J*, effect of added NaCl or KCl (0–500 mM) on M^pro^ cleavage activity on ECFP-C4-ECYP substrate at pH 7.5. Each point represents mean of n = 12 runs with standard deviation shown as error bars. ECFP, enhanced cyan fluorescent protein; EYFP, enhanced yellow fluorescent protein; M^pro^, main protease.

The impact of buffer composition on the activity of M^pro^ was evaluated to identify the optimal assay conditions. ECFP-C4-EYFP was selected as the representative substrate as the C4 cut-site is the most reactive, allowing for a sensitive measure of minor changes in environmental conditions. Initially, the optimal pH for maximum fluorescence (Fig. 3*A*) and M^pro^ activity (Fig. 3*C*) was found to be pH 7.0. However, addition of 150 mM NaCl decreases both FRET intensity (Fig. 3*B*) and M^pro^ activity (Fig. 3*D*), shifting the optimal pH to 7.5. To assess whether the observed impact of varying salt conditions and pH on M^pro^ activity is the product of changes in M^pro^-substrate binding, altered pKa of the catalytic residues or due to destabilization of the tethered fluorophores themselves, equal pH and NaCl buffers assays were performed on free ECFP (Fig. 3, *F* and *G*) and EYFP (Fig. 3, *H* and *I*). Fluorescence wave scans on ECFP show a general indifference to pH and NaCl concentrations in solution, with only pH 5.0 resulting in a significant decrease in fluorescence. EYFP appears more sensitive to environmental conditions, with maximum emission occurring at pH 8.0 and measuredly decreasing at pH 7.0. Interestingly, while salt concentration appears to have minimal effect on EYFP (calculated isoelectric point of 5.78) (59) at pH 7.0 and above, the higher salt concentration appears to have a destabilizing effect at pH 6.0 and pH 5.0, significantly reducing fluorescence. The pH dependence on fluorescence observed here is a well-known property of GFP-derived fluorescent proteins (60, 61), and has been previously exploited to study the pH of subcellular compartments using both ECFP (62) and EYFP (63). The activity of chromophore is dependent on a specific local arrangement of residues that form an intricate network of hydrogen bonds (64, 65, 66). At low pH, protonation of the chromophore in enhanced GFP results in a shift in excitation maximum resulting in decreased emission (64), mirroring the patterns observed in the fluorescent activity of ECFP and EYFP measured in this study.

Next, the effect of a range of salt concentrations on M^pro^ activity, again using ECFP-C4-EYFP substrate, was examined. Figure 3*J* shows that M^pro^ generally tolerates a range of either NaCl or KCl concentrations, from 0 to ∼75 mM, and there was an observable decrease in activity in increasing salt concentrations beyond that range. Additionally, the similar trend between NaCl and KCl indicates that the decrease in activity can be attributed to changing ionic strength rather than a specific ionic effect. Previous studies have reported significantly higher activity at 0 mM NaCl (50); however, it should be noted that here at 0 mM, trace amount of salt are still present, leftover from protein preparation and initial adjustment of the buffer pH. Lastly, the effect of various classes of molecular crowders on M^pro^ activity was examined. Glycerol, dextran, polyethylene glycol 8K (PEG8K) and bovine serum albumin (BSA) were all added in concentrations ranging from 0 to 200 mg/ml to reactions of 100 nM M^pro^ with 10 μM ECFP-C4-EYFP (Fig. 3*E*). Overall, glycerol appears to have minimal impact on activity, with a small decrease in observed rate attributable to increased solution viscosity. Smaller concentrations of the larger mass crowders dextran, PEG8K, or BSA all have a positive effect on rate with 50 mg/ml BSA having the most observable effect, increasing the reaction rate by ∼65%. It is unclear why only the highest concentrations of dextran and PEG8K, 200 mg/ml shows a similar enhancement. It is possible that at such high concentrations and resulting solution viscosity, protein aggregation is favored thereby enhancing the substrate-M^pro^ interaction. Considering these results, a final assay buffer containing 20 mM Hepes pH 7.5 and 150 mM NaCl were chosen for subsequent kinetic assays, in effort to maximize assay sensitivity and more closely mimic physiological conditions.

### M^pro^ substrate steady-state kinetic parameters

**Figure 4:**
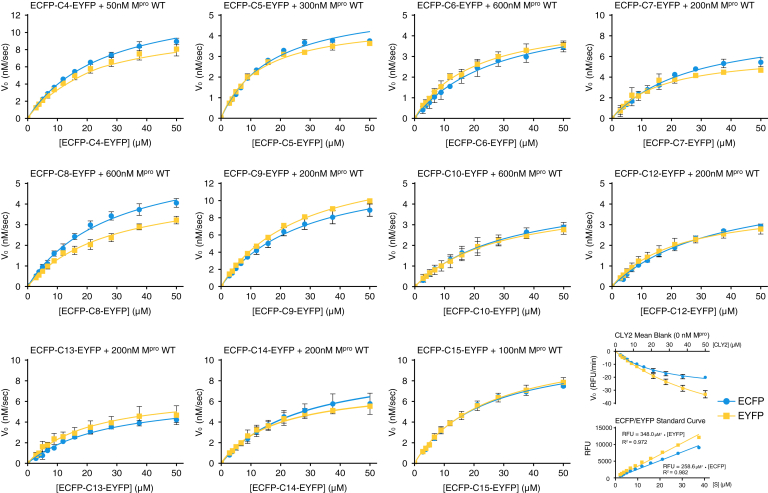
**Michaelis–Menten plots of ECFP-C*x*-EYFP substrate cleavage.** Emission of ECFP (*blue*) and EYFP (*yellow*), measured over 20 min after addition of M^pro^, at 477 nm and 528 nm respectively, *versus* ECFP-C*x*-EYFP substrate concentration. Each curve was subtracted from a blank run in parallel, example average blank shown *bottom right*. Relative fluorescence units were converted to concentration of product over time using constructed ECFP and EYFP standard curves (shown *bottom right*). Each curve was fit using a nonlinear regression to determine *K*_*M*_, *V*_*max*_, *k*_cat_ as summarized in Table 1 alongside *k*_cat_/*K*_*M*_ and quality fit statistics. Each data point is the mean with error bars showing ±1 standard deviation, n = 3. ECFP, enhanced cyan fluorescent protein; EYFP, enhanced yellow fluorescent protein; M^pro^, main protease.

The change in substrate fluorescence at 477 nm (ECFP) and 528 nm (EYFP) over time after addition of M^pro^ was measured as a function of substrate concentration to determine the specificity and reactivity of M^pro^ for viral polyprotein cleavage sequences. Blank reactions of substrate without addition of M^pro^ were included for each substrate series to correct for photobleaching and fluorophore cross-talk (45). Using the initial linear portion (3–20 min) of blank subtracted fluorescence curves to obtain initial velocity (Fig. S6 for example fluorescence over time curves), Michaelis–Menten plots were generated for each substrate (Fig. 4). Initially, the concentration of M^pro^ was kept constant at 50 nM for all experiments; however, the significant differences in M^pro^ reactivity between substrates necessitated higher concentrations of M^pro^ (up to 600 nM for CLY2-C10) to achieve a comparable *V*_*max*_ for the less reactive substrates.

Measurement of the change in fluorescence for both the ECFP donor and EYFP quencher within the FRET system allows for redundancy within equal experimental conditions. However, in our experiments, the observed change in fluorescence of ECFP and EYFP are proportional but not equal, in large part due to the overlap between emission of free ECFP and EYFP at 528 nm (45). The relative contribution to fluorescence at 528 nm for equal concentrations of ECFP and EYFP is approximately 1:3 (as measured, Fig. S5*D*), therefore the recorded emission at 528 nm was adjusted by ∼two-thirds to subtract the contribution of ECFP (67), significantly improving the correlation between ECFP and EYFP curves shown in Figure 4. While small discrepancies remain between pairs of ECFP and EYFP curves, the overall fit of each regression is excellent for all substrates (R^2^ ≥ 0.95) and allows for direct comparison of M^pro^ reactivity between cleavage site positions.

**Figure 5:**
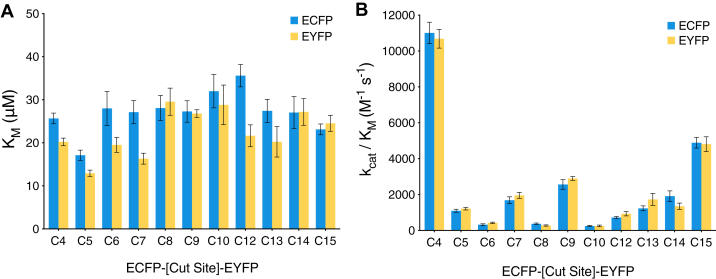
**Substrate affinity and catalytic efficiency of SARS-CoV-2 M**^**pro**^**.** Summary of *K*_*M*_ (*A*) and *k*_cat_/*K*_*M*_ (*B*) values determined from nonlinear regression of Michaelis–Menten plots (Fig. 4) for each ECFP-C*x*-EYFP substrate shown for ECFP in *blue* and EYFP in *yellow* (detailed further in Table 1). Each bar represents mean of n = 3, with error bars showing ±1 standard error of the mean. ECFP, enhanced cyan fluorescent protein; EYFP, enhanced yellow fluorescent protein; M^pro^, main protease; SARS-CoV-2, severe acute respiratory syndrome related coronavirus 2.

In parallel, constructs of either free ECFP or free EYFP modified to mimic the cleavage products of ECFP-C*x*-EYFP (unlinked and without a cut-site sequence; Fig. 1*D* and Table S3) were expressed and purified as per the full-length substrates above. Using these purified fluorophores, standard curves were constructed (Fig. 4) and used to convert relative fluorescence units (RFU/min) to concentration (M^−1^ s^−1^), allowing *k*_cat_ and *k*_cat_/*K*_*m*_ to be calculated for each substrate. Resulting *K*_*m*_ values suggest minimal differences in substrate binding, increasing from 17.1 μM for C5 to 32.1 μM for C10 then trending downward to 23.1 μM for C15 (Fig. 5*A*; comparing values calculated from ECFP curves). However, these differences are not significant enough to infer any true change in equilibrium constant between substrates (68). In contrast, *k*_cat_ values exhibit significant variability across cut-site sequences, which are reflected in the resulting *k*_cat_/*K*_*m*_ values ranging from 11,008 M^−1^ s^−1^ to 249.7 M^−1^ s^−1^ for C4 and C10, respectively (Fig. 5*B* and Table 1). In all experiments C4 was consistently the most efficiently cleaved sequence by a significant margin, with a *k*_cat_/*K*_*m*_ more than double the second most reactive substrate, C15 with a calculated *k*_cat_/*K*_*m*_ of 4888 M^−1^ s^−1^, followed by C9, C7, C14, C13, C12, and C5 (see Table 1 for details). The C6/C8/C10 cleavage sites are the least reactive, with the lowest *k*_cat_/*K*_*m*_ values (<400 M^−1^ s^−1^), each requiring increased concentrations of M^pro^ to measure a consistent change in fluorescence over 20 min. It was also observed that in runs with these less reactive substrates and low concentrations of M^pro^, there was a substantial delay in the expected rate of change of fluorescence from substrate cleavage (not shown). This was likely a direct result of the low reactivity toward these substrates with the diminished initial rates being overwhelmed by the effect of photobleaching on the sample. At the higher concentrations of M^pro^ used subsequently, this effect was no longer observed.

**Table 1:** Steady-state kinetic parameters for SARS-CoV-2 M^pro^ fluorescent cut-site sequence substrates

Cleavage site sequence	ECFP				EYFP			
	k_cat_/K_M_ (M^−1^ s^−1^)	K_M_ (μM)	k_cat_	R^2^	k_cat_/K_M_ (M^−1^ s^−1^)	K_c_ (μM)	k_cat_	R^2^
C4	11,000 ± 600	25.7 ± 1.2	0.2825 ± 0.0071	0.995	10,780 ± 520	20.2 ± 0.9	0.2160 ± 0.0047	0.995
C5	1099 ± 85	17.1 ± 1.2	0.0188 ± 0.0006	0.987	17 ± 75	12.9 ± 0.7	0.0157 ± 0.0004	0.988
C6	322 ± 51	28.0 ± 4.0	0.0090 ± 0.0007	0.958	426 ± 42	19.5 ± 1.7	0.0083 ± 0.0003	0.977
C7	1690 ± 190	27.1 ± 2.7	0.0457 ± 0.0024	0.979	1960 ± 170	16.3 ± 1.3	0.0319 ± 0.0011	0.983
C8	388 ± 40	28.2 ± 2.5	0.0109 ± 0.0005	0.982	296 ± 31	27.8 ± 2.6	0.0082 ± 0.0004	0.981
C9	2560 ± 270	27.3 ± 2.5	0.0699 ± 0.0033	0.981	2900 ± 110	26.8 ± 0.9	0.0777 ± 0.0013	0.998
C10	250 ± 35	32.0 ± 3.9	0.0080 ± 0.0005	0.971	255 ± 46	28.8 ± 4.6	0.0073 ± 0.0006	0.947
C12	722 ± 61	35.6 ± 2.6	0.0257 ± 0.0011	0.991	931 ± 121	21.6 ± 2.5	0.0202 ± 0.0011	0.964
C13	1230 ± 140	27.4 ± 2.7	0.0338 ± 0.0017	0.980	1730 ± 340	20.2 ± 3.5	0.0351 ± 0.0030	0.922
C14	1910 ± 300	27.0 ± 3.7	0.0517 ± 0.0038	0.961	1350 ± 170	27.2 ± 3.1	0.0367 ± 0.0022	0.975
C15	4890 ± 300	23.1 ± 1.3	0.1130 ± 0.0030	0.992	4810 ± 410	24.5 ± 1.9	0.1180 ± 0.0046	0.986

**Figure 6:**
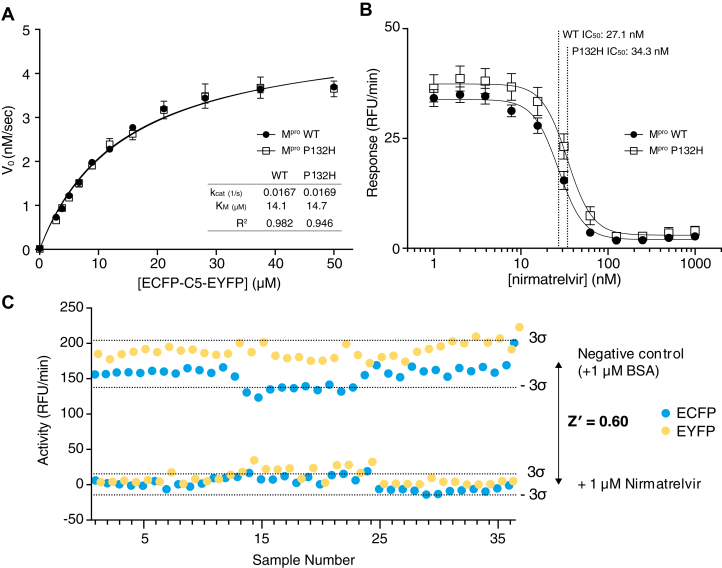
**Effect of P132H mutation in M**^**pro**^**on proteolytic activity and nirmatrelvir inhibition.***A*, Michaelis–Menten plot of ECFP-C4-EYFP substrate with 300 nM M^pro^ WT (*filled circles*; n = 6) or M^pro^ P132H (*squares*; n = 4). In both cases the resulting plots are almost identical with *K*_*M*_ = 14 μM and *k*_cat_= 0.017s^−1^ in both cases. Each point represents mean of runs collected over 20 min, with error bars showing ±1 standard deviation. *B*, response curve of 10 μM ECFP-C4-EYFP substrate (ECFP-C15-EYFP shown in Fig. S7) with 50 nM M^pro^ WT (*filled circles*) or M^pro^ P132H (*squares*) and nirmatrelvir concentrations ranging from 1 nM to1 μM. Calculated IC_50_ from each dose curve shows that while nirmatrelvir is still a potent inhibitor in either case, there is a clear difference in inhibitory effect between WT and P132H M^pro^ variants. Each point represents mean of n = 12 runs collected over 20 min, with error bars showing ±1 standard deviation. *C*, assay quality statistics for SARS-CoV-2 M^pro^ FRET substrates with 25 μM ECFP-C4-EYFP and 300 nM M^pro^ WT. Samples were prepared with 1 μM nirmatrelvir as a positive control for M^pro^ inhibition. An equal concentration BSA was added to a separate set of samples that were otherwise prepared in identical conditions as a negative control. BSA, bovine serum albumin; ECFP, enhanced cyan fluorescent protein; EYFP, enhanced yellow fluorescent protein; M^pro^, main protease; SARS-CoV-2, severe acute respiratory syndrome related coronavirus 2.

To evaluate the suitability of these FRET substrates to probe for changes in the M^pro^ activity and drug inhibition from emerging functional mutations, we compared the reactivity of WT M^pro^ and P132H M^pro^ against the C4 linked substrate, as this mutation in the omicron variant sequence of SARS-CoV-2 is the most prevalent M^pro^ sequence substitution observed to date (https://www.who.int/activities/tracking-SARS-CoV-2-variants) (69). Our results show no discernible differences between the activity of WT and P132H M^pro^ against the C4 cut-site, with near identical values of *k*_cat_ (0.016 s^−1^) and *K*_*m*_ measured (14.7 μM; Fig. 6*A*). Similarly, our FRET results show no significant difference in the inhibition of WT M^pro^ *versus* P132H M^pro^ by nirmatrelvir, with an IC_50_ of 27.1 nM and 34.3 nM, respectively (Fig. 6*B*). Identical trends in P132H activity and inhibition were also observed in repeated assays with the C15 substrate (Fig. S7*A*) and an additional noncovalent, nonpeptide M^pro^ inhibitor, C5a (40) (Fig. S7*B*). To further validate this system for use in high throughput screening of M^pro^ inhibitors, the Z’-factor for inhibition of C4 substrate cleavage was determined. The Z'-factor is a measure for assessing assay quality, considering the signal range difference between positive and negative controls and the consistency of their signal's variability (70). A larger signal range and lower variability indicate a more reliable assay, resulting in a higher Z’-factor. To assess the Z’-factor for the tethered peptide FRET system, the mean and standard deviation of the initial rate was measured using both EYFP and ECFP for 36 positive and 36 negative controls added to samples of ECFP-C4-EYFP and WT M^pro^. Nirmatrelvir was used as a positive control, as a known potent inhibitor of M^pro^ activity (32), and BSA was used as a negative control. As shown in Figure 6*C*, with 1 μM nirmatrelvir added almost no cleavage is observed, with the corresponding BSA controls showing reaction rates ranging from 140 to 200 RFU/min. From this data, independent Z'-factors for ECFP and EYFP were determined to be 0.64 and 0.78, respectively, combining to an overall Z’-factor of 0.60, indicative of an excellent assay for inhibitor screening (70).

## Discussion

In the context of a FRET-based experiment, the tethered peptide substrate system presented here has several advantages over previously reported peptide substrate fragments. Previous FRET substrates used in M^pro^ assays have suffered from poor solubility and large inner filter effects when used at the high concentrations needed to reach saturating substrate concentrations (*V*_*max*_) (50, 71, 72). Consequently, kinetic values for M^pro^ reported using these substrates can vary greatly depending on chemical properties (see Table S2 for summary). Additionally, these previous studies fail to report values for all pp1ab M^pro^ cut-site sequences. Unlike smaller fluorophore activated peptides, alterations in the cleavage-site sequence within the context of the highly soluble ECFP-C*x*-EYFP system have minimal impact on solubility and stability of the various substrate regions embedded within. On a more practical note, tethered FRET pair systems that are protein based are more cost-effective and reproducible compared to canonical peptide substrates, as each cut-site sequence can be easily cloned and recombinantly expressed without the need for additional posttranslation chemical modification. This system may also be suitable for screening of nonnative optimal substrates or ideal binders through an error-prone PCR directed evolution approach (73).

However, these tethered peptide substrates are not without potential drawbacks which may complicate kinetic analysis. In comparison to HPLC-purified small-molecule substrates, the lower stock concentration and purity of ECFP-C*x*-EYFP constructs may prevent saturation of the enzyme required to characterize weakly binding substrates. The assembly of the larger protein substrate itself may also introduce variability, as the polyprotein cleavage sites within the linker may adopt different conformations than those within pp1a and pp1ab. Additionally, the presence of ECFP and EYFP might modulate the binding of the cleavage sites to M^pro^, further complicating accurate enzymatic analysis. Characterization of each cut-site within the context of its native flanking nsp proteins would be ideal; however, the difficulty in expressing full-length pp1a/pp1ab remains an unresolved obstacle. With these considerations in mind, we propose that the tethering of the cleavage site ends to larger proteins is more representative of the environment found in the viral polyprotein and allows for presentation of the substrate sequences in a more consistent manner despite these limitations. Our results show very similar kinetic trends for cut-sites which have been reported in previous studies which used chemically modified peptides (50, 74), particularly for the C4 cut-site which has been the most extensively studied to date (Tables 1 and S2), further validating our approach.

Using this ECFP-C*x*-EYFP system, our analysis shows that M^pro^ *trans* binding affinity for each polyprotein cleavage site position is generally equivalent (Fig. 5*A*), with the maximal difference being observed between C5 and C12 with *K*_*M*_ values of 17.1 ± 1.2 μM and 35.6 ± 2.6 μM, respectively (Table 1). In the context of the physiological polyprotein pp1ab substrate, we suggest C5 having preferential binding within the M^pro^ binding pocket is reasonable as cleavage of nsp5-nsp6 junction allows M^pro^ to first free its own C-terminal tail from the larger polyprotein assembly. Beyond this slight preference for C5, there appears to be little preference in binding among the remaining cut-sites. However, when considering the catalytic efficiency of M^pro^, significant differences emerge between cut-site sequences (Fig. 5*A*). By far, the C4 junction is the most reactive of all polyprotein cleavage site sequences with a *k*_cat_/*K*_*M*_ that is more than double any other position. This reactivity is consistent with previous studies that have also found M^pro^ to be highly active against the nsp4-nsp5 junction (50, 75), which can be attributed to the high similarity between C4 and the preferred A-X-L-Q↓(A/S) cleavage sequence of SARS-CoV-2 M^pro^ as determined by N-terminomics studies (24, 76).

The significantly higher activity of C4 cleavage may act to counterbalance the lower catalytic efficiency of either *cis* or *trans* processing during initial cleavage of M^pro^ protomers bound within the nascent polyprotein. Disruption of M^pro^ dimerization with a P9T mutation lowers catalytic efficiency by >50 fold, highlighting the potential need for a highly reactive site to facilitate efficient liberation of M^pro^ prior to sufficient buildup of the mature dimer and subsequent *trans*-cleavage activity on subsequent cleavage sites. The initial *cis* cleavage at the N terminus of M^pro^ within the polyprotein has previously been assumed due to the spatial proximity of each N terminus to the active site of adjacent M^pro^ protomers, as observed in the crystal structures of the mature enzyme (77, 78, 79). This model is further supported by observations that monomeric forms of M^pro^ exhibit N terminal processing capabilities (80). However, an initial *trans* mechanism in which M^pro^ captive within the polyprotein forms a transient dimeric structure of the mature form, and cleaves the N terminus of another polyprotein molecule has been shown to be sufficiently robust for autoprocessing (77, 81). In either model, the enhanced reactivity of C4 supports efficient release of M^pro^ and formation of the mature active dimer above processing of other nsp-nsp junctions and therefore optimal processing of the viral polyprotein.

Coordination of the polyprotein processing plays a vital role in viral replication (8). This significance was extensively demonstrated in the context of the nsp7–10 region's processing sequence, wherein virus replication was found to be fatally compromised by domain deletions, substitutions, or mutations at cleavage sites (12). Examining the specificity constants *k*_cat_/*K*_*M*_ of the cut-site sequences determined here suggests a clear difference in order of nsp release from pp1a and pp1b. In pp1b, there is a strong preference for C15, then a stepwise decrease from C14 though C12. However, considering only *k*_cat_/*K*_*M*_ values for pp1a sequences, following C4, the suggested cleavage order is C9, followed by C7, C5, C6, and eventually C8. In both cases, this clear preference in cleavage efficiency is not simply the product of the different solubility or chemical properties of the cleavage sites sequences. Calculated aggregation temperature (T_agg_) for each ECFP-C*x*-EYFP substrate used in our study suggests C10 is the most stable, decreasing equally toward C5 and C15, with C4 having a T_agg_ equivalent to C6 and C14 (Fig. S8), a trend that clearly does not align with the observed *k*_cat_/*k*_*M*_ of each sequence.

However, the results from our FRET experiments are in direct contrast with the suggested cleavage order determined by previous hydrogen deuterium exchange mass spectrometry (HDX-MS) analysis and SDS-PAGE proteolytic results of nsp7-11 complex processing (7) by M^pro^. These results conclude the processing order to be: C9 (nsp9-10), C8 (nsp8-9), C10 (nsp10–11), and lastly C7 (nsp7-8). It was proposed that the nsp7-nsp11 polyprotein is dynamic and samples multiple conformations which help to orient the enzyme and substrate for cleavage *via* multiple transient contacts between M^pro^ and the larger complex (7). A cryo-EM structure of catalytically inactive M^pro^ C145A bound to the nsp7-nsp11 complex showed that M^pro^ was exclusively bound to C9 (a preference consistent with our findings), with no observable subpopulations of M^pro^ in complexes with other cut-sites present. Closer examination showed that M^pro^ exclusively forms contacts with the recognition site residues, having minimal interactions with the rest of the polyprotein structure (10).

Extrapolating polyprotein processing order directly from M^pro^ substrate specificity using isolated peptide sequences that mimic polyprotein cleavage points is not possible, as was shown previously for SARS-CoV-1 (7). This is due to the pivotal role of polyprotein subcellular localization (13, 82, 83, 84, 85), in conjunction with nsp conformation and accessibility in governing processing, as was shown for the nsp7-nsp11 complex (7, 10, 12, 15). Likewise, a clear structural determinant of M^pro^ cleavage efficiency is not evident, but this is reflective of the flexibility of M^pro^ toward cleavage targets, requiring only an absolutely conserved glutamine at the P1 position. Comparison of structures of catalytically inactive M^pro^ with cut-site sequences bound shows this promiscuity, with the binding pocket being able to accommodate the variation observed in cleavage site sequences (22, 86) including at alternate binding orientations (21). Structural comparison of a subset sequences which contrast in both steric bulk (small C4/C6 *versus* bulky C10/C15) and cleavage efficiency (high C4/C15 *versus* low C6/C10; Fig. S9) highlights that in each case, the binding surface between each sequence and M^pro^ remains relatively constant despite comparatively large differences in accessible surface area of the free residues (Fig. S9, *B* and *C*; data for all cleavage sequences shown in Table S4). The higher average B-factor (Fig. S9*D*) of the C10 and C6 sequences in these structures may mirror the lower catalytic efficiency observed, but this appears to be independent of both steric bulk and hydrophobicity of the sequences (Fig. S9*E*).

**Figure 7:**
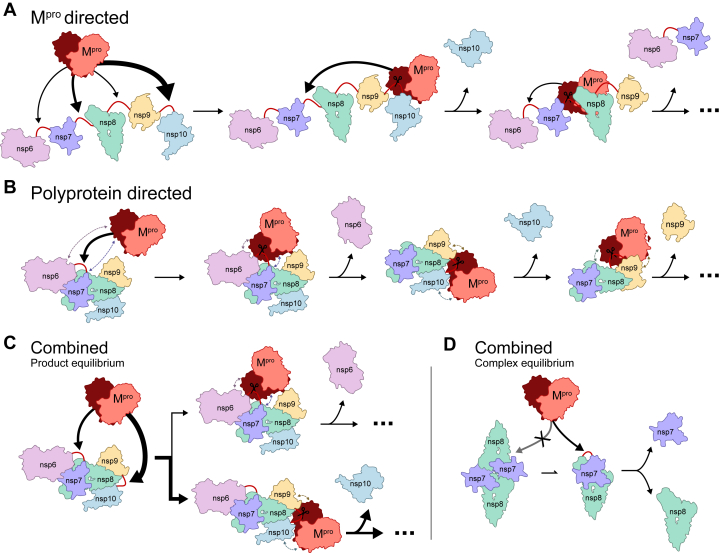
**Polyprotein processing order is governed by the organization and interplay of two biochemical properties of M**^**pro**^**.** Simplified model of different interaction models for polyprotein order processing with *larger arrows* indicating a preference for cleavage. *A*, recognition sites are exposed and M^pro^ dictates order of cleavage based on affinity and catalytic efficiency. *B*, structuring of polyprotein exposes sites and interactions between proteins (*dashed lines*) guides M^pro^ cleavage. Combination of both modes of action allows for tighter control of both (*C*) product equilibrium and (*D*) control of complex lifetimes. Note that exact order of processing shown here is illustrative only and not an accurate summary of the intricate balance of complex formation and product release noted by previous studies (7, 10, 108, 109, 110). M^pro^, main protease.

Two models of polyprotein processing control have been previously proposed (10). In the first “M^pro^ directed” model (Fig. 7*A*) the cut-sites along the polyprotein are exposed on the surface of the polyprotein for recruiting M^pro^ at the primary cleavage sites. This is akin to the beads on a string conformation, where the affinity and rate of cleavage of each cut-site sequence determine the order of cleavage, with the most reactive sites being processed first. This model is supported by the minimal interaction noted in the M^pro^ cryo-EM structure (10), where M^pro^ engages only with the recognition site residues and does not interact with the polyprotein to a significant extent. HDX-MS experiments on the nsp7-10 complex showed high levels of solvent exchange at each cut-site, consistent with cleavage regions that are accessible for processing (7). This model would therefore suggest that the polyprotein is processed in the order of reactivity determined here (Fig. 7*B*).

In the second “polyprotein directed” model, the order of processing is governed nsp complex quaternary structure, interprotein contacts (outside of the M^pro^ cleavage site) and cleavage site accessibly (Fig. 7*B*). This model would reconcile the difference in the order of cleavage of the nsp7-10 complex observed in by SDS-PAGE (10) and HDX-MS experiments (7), *versus* the catalytic efficiencies determined here. Further structural analysis shows that C9, which is the first site to be processed in the nsp7-10 complex, was the most exposed junction and typically adopts a random coil, potentially facilitating interaction with M^pro^. In contrast, C7, the last site to undergo cleavage, was more obscured and mostly adopted an α-helical conformation possibly interfering with effective cleavage (7).

However, it remains unlikely that this polyprotein directed model is the sole contribution in determining polyprotein processing. Given that cleavage site sequences are highly conserved between SARS-CoV-2 variants, and the key motifs are conserved between different coronaviruses, there must be a mechanistic advantage for the observed distribution of cut-site sequences and their associated cleavage kinetics. For example, the FYP(K/R/Q) motif conserved in C15 (Fig. S1) could be a determinant in the higher activity of M^pro^ toward C15 (Fig. 5*B*), and reflective of the priority of separating nsp15 and nsp16 during the lifecycle of SARS-CoV-2. Therefore, while it is likely that access to each cleavage site is governed by polyprotein structuring, there is still a preference for M^pro^ to process exposed cleavage sites at different rates. We propose a combined model, with the interplay between cut-site accessibility and differences in the catalytic efficiency observed here allowing for tighter regulatory control of nsp stoichiometry (Fig. 7*C*) and intermediate nsp complex formation (Fig. 7*D*) (15, 87), both of which have been shown to have critical roles in the viral life cycle as in the case of RNA-dependent RNA polymerase formation and regulation (88, 89). This model may also help to explain the detection of uncleaved C6, C8, and C10 after overnight incubation with M^pro^ (Fig. S4), as the lower reactivity of these cut-sites may facilitate an equilibrium of nsp-nsp complexes *versus* free monomers, although the exact relationship between these forms remains to be studied in greater detail.

This intricate regulation of intermediate complexes and nsp release could also explain why so few mutations within the binding cleft of M^pro^ have been observed in clinical variants, despite reports of engineered mutations which confer increased resistance to protease inhibitors (primarily nirmatrelvir) yet conserve M^pro^ activity (90). To accommodate such a diverse combination of residues, the binding cleft of M^pro^ is known to have a high degree of plasticity, enabling remarkable flexibility within the substrate specificity binding pockets surrounding the target scissile bond (91) (see Chapter 2). *In vitro* mutational analysis has shown that outside of a few critical motifs (primarily within the binding cleft and dimerization interface), M^pro^ is remarkably tolerant to point mutations, with most variants retaining WT-like function (38). However, M^pro^ from SARS-CoV-1 and SARS-CoV-2 retain 96% sequence similarity (91, 92, 93) and a few functional mutations have been observed in arising clinical strains to date (94) (less than 0.4% of clinical variants having two or more M^pro^ mutations as cataloged in the CoV-Glue-Viz database (95), Global Initiative on Sharing All Influenza Data (GISAID's) human archive of SARS-CoV-2 sequences) (96).

All of the covalent interactions between M^pro^ and its cleavage sequences occur within the S1 subsite and the bulk of the remaining noncovalent interactions are encompassed by the P2-P2’ positions. Our results show that the binding equilibrium between each cut-site is relatively equal (Fig. 5*A*) yet maintain pronounced differences in reactivity (Fig. 5*B*). Therefore, mutations outside of the main S1 subsite (and S1ʹ/S2 to a lesser degree) that are not outright detrimental to substrate binding can confer resistance to inhibitor binding and maintain overall protease activity. However, these variants may still interfere with the delicate balance of nsp (and nsp complex) processing and therefore the lifecycle of the virus within the cell, possibly explaining why these resistance mutations have yet to emerge in clinical variants.

M^pro^ remains an important drug target for combatting SARS-CoV-2 infection, and various potent inhibitors have emerged by exploiting the binding envelope and kinetics of M^pro^ proteolysis as determined through extensive structural and kinetic studies (27, 28, 32, 40). Additionally, fully characterizing M^pro^ kinetics and polyprotein processing has broader implications for understanding the many other +ssRNA viruses that use a polyprotein genomic organization strategy. Using a tethered peptide FRET-based system that avoids common pitfalls associated with previously published kinetic studies of M^pro^, we characterized SARS-CoV-2 M^pro^ activity and determined the steady-state kinetic parameters of all eleven polyprotein cleavage sequences. Screening the interactions of other viral proteases against their native cut-site sequences, particularly other coronaviruses for comparison, could further highlight the interplay between cut-site sequence composition and polyprotein processing. We also show that this FRET system is sensitive yet readily adaptable to study the effect of mutations within M^pro^ on cut-site cleavage or characterize M^pro^ inhibitors in high throughput screening assays (as shown in Figs. 6 and S7). Using this system, the impact of mutations, particularly from emerging variants, could reveal changes (or lack thereof) in polyprotein cut-site cleavage and by extension differences in the viral intracellular response.

## Experimental procedures

### Cloning, protein production, and purification of M^pro^

The gene encoding full-length SARS-CoV-2 M^pro^ (UniProt P0DTD1) was cloned into a modified pET28a plasmid including an N-terminal dual His-SUMO tag to facilitate expression and purification (21). Mutant P132H M^pro^ was generated using QuickChange site-directed mutagenesis on the same pET28a His-SUMO plasmid. *E. coli* BL21 (DE3) were transformed with the pET28a His-SUMO plasmid *via* electroporation. Cells were grown at 37 °C in LB media supplemented with 0.05 mg/ml kanamycin. At an *A*_600_ of ∼1, protein expression was induced with the addition of IPTG to a final concentration of 1 mM and the expression temperature was lowered to 16 °C. Cells were harvested after 5 h, resuspended in lysis buffer (50 mM Tris pH 8, 300 mM NaCl, 1% Triton-X100, 10 mM MgCl_2_, 0.01 mg/ml DNase 1), and lysed *via* sonication while incubating on ice. The lysate was centrifuged at 50,000*g* for 60 min, and the soluble protein was loaded onto a gravity flow column packed with 5 ml HisPur Ni-NTA resin (Thermo Fisher Scientific) equilibrated in purification buffer (20 mM Hepes pH 8, 300 mM NaCl) containing 20 mM imidazole. The column was washed with five column volumes of the buffer with 50 mM imidazole, and then eluted with purification buffer containing 300 mM imidazole. Elution fractions containing M^pro^ were combined, concentrated, and buffer exchanged during concentration with Amicon Ultra centrifugal filter (30 kDa molecular weight cut-off [MWCO]) at 4000 rpm. The sample was concentrated to ∼ 10 ml in 50 mM Hepes (pH 8), 300 mM NaCl, 5 mM BME and incubated with 20 μg/ml SUMO protease overnight at 4 °C with gentle agitation to cleave the N-terminal H-SUMO tag. Uncleaved His-SUMO-M^pro^, cleaved His-SUMO tag, and His-tagged SUMO protease were all removed by 5 ml HisPur Ni-NTA resin as above. The flowthrough and wash fractions containing cleaved M^pro^ were collected and further concentrated to 1 ml with an Amicon Ultra centrifugal filters (30 kDa MWCO) before further purification by gel-filtration chromatography with a Superdex 200 Increase 5/150 Gl column (Cytiva) equilibrated in 20 mM Hepes pH 7.5, 150 mM NaCl, 1 mM EDTA, 1 mM DTT. Pooled peak fractions containing M^pro^ were concentrated by ultrafiltration (Amicon Ultra centrifugal filter; 30 kDa MWCO) to >10 mg/ml and frozen in liquid nitrogen for storage at −80 °C. Final concentration was determined by absorbance at 280 nm using the extinction coefficient of 32,890 M^−1^ cm^−1^ calculated from the primary sequence of the construct (59).

### Cloning, protein production, and purification of ECFP-C*x-*EYFP constructs

The gene encoding CLY2 was obtained from Addgene (Addgene plasmid # 21761; http://n2t.net/addgene:21761; RRID:Addgene_21761). Eleven sets of three primers (Table S3) were used to generate inserts that contained each polyprotein cut-site (C4-C10, C12-C15; Fig. 1*D* for detailed sequences) flanked by GGSGGS repeats and EcoRI and NcoI restriction sites. These inserts were then digested with NocI-HF and EcoRI-HF (New England Biolabs) alongside pET28CLY2 prior to ligation, purification, and transformation of *E.coli* DH10b. The plasmid was isolated from DH10b cultures using a mini-prep plasmid purification kit (iNtRON Biotechnology) prior to sequencing to confirm cut-site insertion.

*E. coli* BL21 (DE3) were transformed with each pET28 H-Th-ECFP-C*x*-EYFP plasmid *via* electroporation. Cells were grown at 37 °C in LB media supplemented with 0.05 mg/ml kanamycin. At an *A*_600_ of ∼1, protein expression was induced with the addition of IPTG to a final concentration of 1 mM, and the temperature lowered to 20 °C for expression overnight. Cells were harvested after ∼16 h, resuspended in lysis buffer (50 mM Tris pH 8, 300 mM NaCl, 1% Triton-X100, 10 mM MgCl_2_, and 0.01 mg/ml DNase 1), and lysed *via* sonication while incubating on ice. The lysate was centrifuged at 50,000*g* for 60 min, and the soluble protein was loaded onto a gravity-flow column packed with 5 ml HisPur Ni-NTA resin (Thermo Fisher Scientific) equilibrated in purification buffer (20 mM Hepes pH 7.5 and 150 mM NaCl) containing 20 mM imidazole. The column was washed with five column volumes of the buffer with 50 mM imidazole, and then eluted with purification buffer containing 300 mM imidazole. Subsequently, 10 μg of bovine alpha-thrombin protease (Prolytix) was added to the pooled elution fractions containing ECFP- C*x*-EYCP and dialyzed against 2L purification buffer overnight to remove the N-terminal His_6_ expression tag. Cleaved ECFP- C*x*-EYCP was isolated with 5 ml HisPur Ni-NTA resin as above, but the flowthrough and wash fractions collected instead. The pooled fractions containing ECFP- C*x*-EYCP were further concentrated to 3 ml with an Amicon Ultra centrifugal filter (30 kDa MWCO) before further purification by gel filtration chromatography with a Superdex 200 Increase 5/150 GL column (Cytiva) equilibrated in 20 mM Hepes pH 7.5, 150 mM NaCl, 1 mM EDTA, and 1 mM DTT. Pooled peak fractions containing ECFP- C*x*-EYCP were concentrated by ultrafiltration (Amicon Ultra Centrifugal Filter; 30 kDa MWCO) to 500 nM and frozen in liquid nitrogen for storage at −80 °C. Final concentration was determined by absorbance at 280 nm using the extinction coefficient of 49,530 M^−1^ cm^−1^ calculated from the primary sequence of the construct (59).

To generate the ECFP and EYFP standard curves, two fusion protein constructs were created to mimic the cleavage product of the reaction between ECFP-C*x*-EYFP and. His_6_-Th-ECFP-(GGSGGS) and (GGSGGS)-EYFP-Th-His_6_ were cloned using a restriction-free method to remove either EYFP or ECFP regions from CLY2 to leave a single fluorophore and half of the disordered linker. For the (GGSGGS)-EYFP construct a Th-His_6_ expression tag was added to facilitate purification. These two constructs were then transformed and expressed as above, with only minor modifications to accommodate for differences in molecular weight between ECFP-C*x*-EYFP and free ECFP/EFYP (∼56 kDa *versus* ∼27 kDa, respectively).

### SDS-PAGE and densitometry analysis

All SDS-PAGE experiments were run with 15% acrylamide gels and visualized with Coomassie brilliant blue G staining prior to imaging with a Bio-Rad Gel Doc EZ imager. For cleavage time trials, 50 μM ECFP-C4-EYFP was incubated with 1 μM M^pro^ at room temperature and samples were taken at regular intervals, mixing with SDS loading dye to stop the reaction prior to loading. Densitometry analysis was performed using Bio-Rad image lab (v6.1.0; bio-rad.com/product/image-lab-software) quantity tools, using fixed amounts of uncleaved controls and GangNam-STAIN Prestained Protein Ladder (iNtRON Biotechnology) as internal standards. All values were then processed and plotted in Microsoft Excel to generate representative plots.

### Enzyme assay general methods

All reactions were run in black 96-well flat-bottom polypropylene microplate (Greiner Bio-One; ref 655209) with a sample volume of 150 μl per well. Fluorescence was measured using a BioTek Synergy H4 microplate reader controlled by BioTek Gen5 software (agilent.com/en/product/cell-analysis/cell-imaging-microscopy/cell-imaging-microscopy-software/biotek-gen5-software-for-imaging-microscopy-1623226). All experiments were conducted at 25 °C, in the same buffer used during final purification of each substrate, 20 mM Hepes pH 7.5, 150 mM NaCl, 1 mM EDTA, and 1 mM DTT. An excitation wavelength of 434 nm (slit with of 9 nm) was used, and emission wavelengths of 477 nm and 528 nm were recorded with a slit with of 9 nm unless otherwise noted. Each data point was the sum of 30 measurements taken from a read height of 8 mm after 100 msec delay when switching between wells. Between each round of measurement, plates were automatically agitated with gentle shaking for 5 s to minimize the effect of localized photobleaching. The screening of buffer conditions on M^pro^ activity used equal setup and conditions, with exceptions noted where applicable (Fig. 3).

### Steady-state enzyme kinetics

Initial emission wave scan experiments were conducted by measuring the emission intensity from 450 nm to 550 nm (in 2.5 nm steps) of 10 μM ECFP-C*x*-EYFP after excitation at 434 nm. To determine activity of M^pro^ against each FRET substrate, 10 μM ECFP-C*x*-EYFP was incubated at room temperature overnight with 100 nM M^pro^ prior to collecting an emission wave scan, which was then compared to an identical sample similarly incubated overnight without added M^pro^. These results were visualized using the ggplot2 (97; ggplot2.tidyverse.org) package in R (98; r-project.org) and are summarized in Figure 2.

To evaluate initial reaction rates, emission readings at 434 nm and 528 nm were sampled every 80 s over 4 h to measure complete hydrolysis of 25 μM of substrate with 50 nM of added M^pro^ alongside SDS-PAGE densitometry analysis (Fig. S6). For later kinetic assays, measurements were taken every 25 s over 20 min, and the initial rates of reaction (*v*_0_), collected in triplicate at each substrate concentration, were fit to the linear portion of the reaction progress (3–20 min of each run) corresponding to less than 10% substrate hydrolysis. For enzymological characterization the final concentration of M^pro^ ranged from 50 nM to 600 nM depending on substrate reactivity, while that of the substrate consistently spanned the range between 2.8 μM and 50 μM, with 0 μM substrate included in each run as a control.

For each concentration of ECFP-C*x*-EYFP substrate, the baseline change in fluorescence of the substrate in the absence of enzyme was subtracted from the observed change in fluorescence with enzyme. Given the contribution of ECFP to fluorescence at 528 nm, the recorded emission at 528 nm was adjusted by two-thirds to subtract the contribution of ECFP from the signal from EYFP fluorescence (determined by comparing emission at 528 nm of equal amounts of free ECFP and EYFP after excitation with either 434 nm or 514 nm light; summarized in Fig. S5*D*). After correcting these values for photobleaching by blank subtraction, the inner filter effect, and converting to units of cleaved product as a function of time (*i.e.*, M/s) using a calibration curve constructed with prepared free ECFP or EYFP (Fig. 4 for example standard curves). A plot of reaction rate in M/s *versus* the molar substrate concentration was fit to the Michaelis–Menten equation to obtain values of *K*_*M*_ and *V*_*max*_ using the non-linear, least squares regression analysis in Graphpad Prism 9 software (GraphPad Software; graphpad.com). To calculate *k*_cat_, *V*_*max*_ was divided by the molar concentration of enzyme used in each assay (as determined above). With these values of *k*_cat_ and *K*_*M*_, the value of *k*_cat_/*K*_*M*_ was subsequently calculated assuming a fixed amount of active enzyme used in the experiment.

Similarly, the dose-dependent inhibition of enzyme activity by the inhibitor nirmatrelvir (PF-07321332) and C5a (additional noncovalent, nonpeptide M^pro^ inhibitor) (40) was assayed to validate our FRET system for inhibition studies. In this inhibition assay, performed in triplicate, either WT or P132H was incubated with 1 nM to 1 μM (0 μM also included as a noninhibited control) of nirmatrelvir for 15 min before mixing with ECFP-C4-EYFP substrate to monitor the residual activity. The final enzyme and substrate concentrations were 50 nM and 10 μM, respectively. The linear portion of each emission curve was used (3–20 min) after blank subtraction, and IC_50_ values were determined using a nonlinear, variable slope does-response model in Graphpad Prism 9 software:

$$Y={\;\text{Response}\;}_{\min}\;+\;\frac{{\;\text{Response}\;}_{\max}\;–\;{\;\text{Response}\;}_{\min}}{1\;+\;10^{\left(\text{LogIC}50\;-\;X\right)*\text{HillSlope}}}$$

### Assay quality assessment

The Z’-factor (70) for screening inhibitors was assessed by measuring 36 replicates of enzyme activity with a positive and negative control using the ECFP-C4-EYFP FRET substrate. nirmatrelvir (PF-07321332) added to 1 μM final concentration was used as a positive inhibitor control and 1 μM BSA was used as a negative control. Each reaction contained 25 μM ECFP-C4-EYFP and 300 nM WT M^pro^, in assay buffer (20 mM Hepes pH 7.5, 150 mM NaCl, 1 mM EDTA, and 1 mM DTT). For each assay, the mean and standard deviation of the initial rate for positive and negative controls were calculated. The signal dynamic range was calculated for this M^pro^ mock high-throughput screening assay as per Zhang *et al.* (1999) (70) where μ_c+_ and μ_c-_ are the mean of the negative and positive controls, respectively:

$$\text{Signal}\;\text{dynamic}\;\text{range}={\bar{\mu }}_{c+}-\;{\bar{\mu }}_{c-}$$

The Z’-factor was then calculated where σ_c+_ and σ_c-_ are the standard deviation of the positive and negative controls, respectively:

$$Z^{\prime }=1\;-\;\frac{\left({3\sigma }_{c+}\;+\;{3\sigma }_{c-}\right)}{\left|{\bar{\mu }}_{c+}-\;{\bar{\mu }}_{c-}\right|}$$

### Differential static light scattering

The aggregation temperature (T_agg_) of each ECFP-C*x*-EYFP substrate was determined using the Stargazer-2 differential light scattering platform (Epiphyte3). Using the same buffer conditions as used in kinetic assays (20 mM Hepes pH 7.5, 150 mM NaCl, 1 mM DTT, and 1 mM EDTA), 10 μl dilutions of 2.5 μM to 40 μM substrate was loaded into wells (5 replicates of each concentration per plate) of a black 384 well polystyrene microplate (Corning). Mineral oil (11 μl) was added to each well to prevent evaporation. The Stargazer-2 was then used to perform a temperature scan experiment in triplicate and the resulting T_agg_ values for each substrate concentration were averaged together and visualized using the ggplot2 (97) package in R (98).

### Mass spectrometry

MALDI-TOF mass spectrometry experiments were performed on ECFP-C4-EYFP, ECFP-C6-EYFP, and ECFP-C15-EYFP to validate substrate identity and purity. Samples were prepared in a sinapinic acid matrix, and spectra were collected on a Bruker Autoflex Speed LRF running in linear positive mode. Resulting spectra were processed with smoothing, peak picking by centroid, and internally calibrated using BSA peaks at 333216.0, 66431.0, and 113275.1 to improve mass accuracy.

## Data availability

The data that support this study are available from the corresponding authors upon request.

## Supporting information

This article contains supporting information (15, 21, 22, 28, 29, 30, 40, 41, 46, 47, 48, 50, 71, 74, 79, 86, 99, 100, 101, 102, 103, 104, 105).

## Conflict of interest

The authors declare that they have no conflicts of interest with the contents of this article.

## References

[1] Wu F., Zhao S., Yu B., Chen Y.M., Wang W., Song Z.G. (2020). A new coronavirus associated with human respiratory disease in China. Nature.

[2] Guo Y.-R., Cao Q.D., Hong Z.S., Tan Y.Y., Chen S.D., Jin H.J. (2020). The origin, transmission and clinical therapies on coronavirus disease 2019 (COVID-19) outbreak – an update on the status. Mil. Med. Res..

[3] Atkins J.F., Loughran G., Bhatt P.R., Firth A.E., Baranov P.V. (2016). Ribosomal frameshifting and transcriptional slippage: from genetic steganography and cryptography to adventitious use. Nucleic Acids Res..

[4] Masters P.S. (2006). The molecular biology of coronaviruses. Adv. Virus Res..

[5] Emmott E., de Rougemont A., Hosmillo M., Lu J., Fitzmaurice T., Haas J. (2019). Polyprotein processing and intermolecular interactions within the viral replication complex spatially and temporally control norovirus protease activity. J. Biol. Chem..

[6] Kräusslich H.G., Nicklin M.J., Lee C.K., Wimmer E. (1988). Polyprotein processing in picornavirus replication. Biochimie.

[7] Yadav R., Courouble V.V., Dey S.K., Harrison J.J.E.K., Timm J., Hopkins J.B. (2022). Biochemical and structural insights into SARS-CoV-2 polyprotein processing by Mpro. Sci. Adv..

[8] Sawicki S.G., Sawicki D.L., Younker D., Meyer Y., Thiel V., Stokes H. (2005). Functional and genetic analysis of coronavirus replicase-transcriptase proteins. PLoS Pathog..

[9] Spall V.E., Shanks M., Lomonossoff G.P. (1997). Polyprotein processing as a strategy for gene expression in RNA viruses. Semin. Virol..

[10] Narwal M., Armache J.-P., Edwards T.J., Murakami K.S. (2023). SARS-CoV-2 polyprotein substrate regulates the stepwise Mpro cleavage reaction. J. Biol. Chem..

[11] Shin G., Yost S.A., Miller M.T., Elrod E.J., Grakoui A., Marcotrigiano J. (2012). Structural and functional insights into alphavirus polyprotein processing and pathogenesis. Proc. Natl. Acad. Sci. U. S. A..

[12] Deming D.J., Graham R.L., Denison M.R., Baric R.S. (2007). Processing of open reading frame 1a replicase proteins nsp7 to nsp10 in murine hepatitis virus strain A59 replication. J. Virol..

[13] Bost A.G., Carnahan R.H., Lu X.T., Denison M.R. (2000). Four proteins processed from the replicase gene polyprotein of mouse hepatitis virus colocalize in the cell periphery and adjacent to sites of virion assembly. J. Virol..

[14] Ziebuhr J., Siddell S.G. (1999). Processing of the human coronavirus 229E replicase polyproteins by the virus-encoded 3C-like proteinase: identification of proteolytic products and cleavage sites common to pp1a and pp1ab. J. Virol..

[15] Krichel B., Falke S., Hilgenfeld R., Redecke L., Uetrecht C. (2020). Processing of the SARS-CoV pp1a/ab nsp7-10 region. Biochem. J..

[16] Snijder E.J., Bredenbeek P.J., Dobbe J.C., Thiel V., Ziebuhr J., Poon L.L.M. (2003). Unique and conserved features of genome and proteome of SARS-coronavirus, an early split-off from the coronavirus group 2 lineage. J. Mol. Biol..

[17] Hegyi A., Ziebuhr J. (2002). Conservation of substrate specificities among coronavirus main proteases. J. Gen. Virol..

[18] Hsu M.-F., Kuo C.J., Chang K.T., Chang H.C., Chou C.C., Ko T.P. (2005). Mechanism of the maturation process of SARS-CoV 3CL protease. J. Biol. Chem..

[19] Cheng S.-C., Chang G.-G., Chou C.-Y. (2010). Mutation of Glu-166 blocks the substrate-induced dimerization of SARS coronavirus main protease. Biophys. J..

[20] Gonzalez L.S., Anson B., Mesecar A. (2017). Effect of allosteric changes in MERS 3CL protease enzymatic activity and dimerization. FASEB J..

[21] Lee J., Kenward C., Worrall L.J., Vuckovic M., Gentile F., Ton A.T. (2022). X-ray crystallographic characterization of the SARS-CoV-2 main protease polyprotein cleavage sites essential for viral processing and maturation. Nat. Commun..

[22] Zhao Y., Zhu Y., Liu X., Jin Z., Duan Y., Zhang Q. (2022). Structural basis for replicase polyprotein cleavage and substrate specificity of main protease from SARS-CoV-2. Proc. Natl. Acad. Sci. U. S. A..

[23] Meyer B., Chiaravalli J., Gellenoncourt S., Brownridge P., Bryne D.P., Daly L.A. (2021). Characterising proteolysis during SARS-CoV-2 infection identifies viral cleavage sites and cellular targets with therapeutic potential. Nat. Commun..

[24] Koudelka T., Boger J., Henkel A., Schönherr R., Krantz S., Fuchs S. (2021). N-terminomics for the identification of in vitro substrates and cleavage site specificity of the SARS-CoV-2 main protease. Proteomics.

[25] Jagdeo J.M., Dufour A., Klein T., Solis N., Kleifeld O., Kizhakkedathu J. (2018). N-terminomics TAILS identifies host cell substrates of poliovirus and coxsackievirus B3 3C proteinases that modulate virus infection. J. Virol..

[26] Ullrich S., Nitsche C. (2020). The SARS-CoV-2 main protease as drug target. Bioorg. Med. Chem. Lett..

[27] Zhang L., Lin D., Sun X., Curth U., Drosten C., Sauerhering L. (2020). Crystal structure of SARS-CoV-2 main protease provides a basis for design of improved α-ketoamide inhibitors. Science.

[28] Ma C., Sacco M.D., Hurst B., Townsend J.A., Hu Y., Szeto T. (2020). Boceprevir, GC-376, and calpain inhibitors II, XII inhibit SARS-CoV-2 viral replication by targeting the viral main protease. Cell Res..

[29] Jin Z., Du X., Xu Y., Deng Y., Liu M., Zhao Y. (2020). Structure of Mpro from SARS-CoV-2 and discovery of its inhibitors. Nature.

[30] Dai W., Zhang B., Jiang X.M., Su H., Li J., Zhao Y. (2020). Structure-based design of antiviral drug candidates targeting the SARS-CoV-2 main protease. Science.

[31] Qiao J., Li Y.S., Zeng R., Liu F.L., Luo R.H., Huang C. (2021). SARS-CoV-2 Mpro inhibitors with antiviral activity in a transgenic mouse model. Science.

[32] Owen D.R., Allerton C.M.N., Anderson A.S., Aschenbrenner L., Avery M., Berritt S. (2021). An oral SARS-CoV-2 Mpro inhibitor clinical candidate for the treatment of COVID-19. Science.

[33] Hoffman R.L., Kania R.S., Brothers M.A., Davies J.F., Ferre R.A., Gajiwala K.S. (2020). Discovery of ketone-based covalent inhibitors of coronavirus 3CL proteases for the potential therapeutic treatment of COVID-19. J. Med. Chem..

[34] Namchuk M.N. (2021). Early returns on small molecule therapeutics for SARS-CoV-2. ACS Infect. Dis..

[35] Vuong W., Khan M.B., Fischer C., Arutyunova E., Lamer T., Shields J. (2020). Feline coronavirus drug inhibits the main protease of SARS-CoV-2 and blocks virus replication. Nat. Commun..

[36] Baker J.D., Uhrich R.L., Kraemer G.C., Love J.E., Kraemer B.C. (2021). A drug repurposing screen identifies hepatitis C antivirals as inhibitors of the SARS-CoV2 main protease. PLoS One.

[37] Fu L., Ye F., Feng Y., Yu F., Wang Q., Wu Y. (2020). Both Boceprevir and GC376 efficaciously inhibit SARS-CoV-2 by targeting its main protease. Nat. Commun..

[38] Agost-Beltrán L., de la Hoz-Rodríguez S., Bou-Iserte L., Rodríguez S., Fernández-de-la-Pradilla A., González F.V. (2022). Advances in the development of SARS-CoV-2 Mpro inhibitors. Molecules.

[39] Edwards A. (2020). What are the odds of finding a COVID-19 drug from a lab repurposing screen?. J. Chem. Inf. Model..

[40] Pérez-Vargas J., Worrall L.J., Olmstead A.D., Ton A.T., Lee J., Villanueva I. (2023). A novel class of broad-spectrum active-site-directed 3C-like protease inhibitors with nanomolar antiviral activity against highly immune-evasive SARS-CoV-2 Omicron subvariants. Emerg. Microbes Infect..

[41] Dražić T., Kühl N., Leuthold M.M., Behnam M.A.M., Klein C.D. (2021). Efficiency improvements and discovery of new substrates for a SARS-CoV-2 main protease FRET assay. SLAS Discov..

[42] Coelho C., Gallo G., Campos C.B., Hardy L., Würtele M. (2020). Biochemical screening for SARS-CoV-2 main protease inhibitors. PLoS One.

[43] Jo S., Kim S., Kim D.Y., Kim M.-S., Shin D.H. (2020). Flavonoids with inhibitory activity against SARS-CoV-2 3CLpro. J. Enzyme Inhib. Med. Chem..

[44] Nguyen A.W., Daugherty P.S. (2005). Evolutionary optimization of fluorescent proteins for intracellular FRET. Nat. Biotechnol..

[45] Piston D.W., Kremers G.-J. (2007). Fluorescent protein FRET: the good, the bad and the ugly. Trends Biochem. Sci..

[46] Breidenbach J., Lemke C., Pillaiyar T., Schäkel L., Al Hamwi G., Diett M. (2021). Targeting the main protease of SARS-CoV-2: from the establishment of high throughput screening to the design of tailored inhibitors. Angew. Chem. Int. Ed..

[47] Zhu W., Xu M., Chen C.Z., Guo H., Shen M., Hu X. (2020). Identification of SARS-CoV-2 3CL protease inhibitors by a quantitative high-throughput screening. ACS Pharmacol. Transl. Sci..

[48] Li Z., Li X., Huang Y.Y., Wu Y., Liu R., Zhou L. (2020). Identify potent SARS-CoV-2 main protease inhibitors via accelerated free energy perturbation-based virtual screening of existing drugs. Proc. Natl. Acad. Sci. U. S. A..

[49] Ma C., Wang J. (2021). Dipyridamole, chloroquine, montelukast sodium, candesartan, oxytetracycline, and atazanavir are not SARS-CoV-2 main protease inhibitors. Proc. Natl. Acad. Sci. U. S. A..

[50] Legare S., Heide F., Bailey-Elkin B.A., Stetefeld J. (2022). Improved SARS-CoV-2 main protease high-throughput screening assay using a 5-carboxyfluorescein substrate. J. Biol. Chem..

[51] Anton D.B., Galvez Bulhões Pedreira J., Zvirtes M.L., Laufer S.A., Ducati R.G., Goettert M. (2023). Targeting SARS-CoV-2 main protease (MPro) with kinase inhibitors: a promising approach for discovering antiviral and anti-inflammatory molecules against SARS-CoV-2. J. Chem. Inf. Model..

[52] Ting A.Y., Kain K.H., Klemke R.L., Tsien R.Y. (2001). Genetically encoded fluorescent reporters of protein tyrosine kinase activities in living cells. Proc. Natl. Acad. Sci. U. S. A..

[53] Cicchetti G., Biernacki M., Farquharson J., Allen P.G. (2004). A ratiometric expressible FRET sensor for phosphoinositides displays a signal change in highly dynamic membrane structures in fibroblasts. Biochemistry.

[54] Pearce L.L., Gandley R.E., Han W., Wasserloos K., Stitt M., Kanai A.J. (2000). Role of metallothionein in nitric oxide signaling as revealed by a green fluorescent fusion protein. Proc. Natl. Acad. Sci. U. S. A..

[55] Kohn J.E., Plaxco K.W. (2005). Engineering a signal transduction mechanism for protein-based biosensors. Proc. Natl. Acad. Sci. U. S. A..

[56] Mitra R.D., Silva C.M., Youvan D.C. (1996). Fluorescence resonance energy transfer between blue-emitting and red-shifted excitation derivatives of the green fluorescent protein. Gene.

[57] Felber L.M., Cloutier S.M., Kündig C., Kishi T., Brossard V., Jichlinski P. (2004). Evaluation of the CFP-substrate-YFP system for protease studies: advantages and limitations. BioTechniques.

[58] Evers T.H., van Dongen E.M.W.M., Faesen A.C., Meijer E.W., Merkx M. (2006). Quantitative understanding of the energy transfer between fluorescent proteins connected via flexible peptide linkers. Biochemistry.

[59] Gasteiger E., Hoogland C., Gattiker A., Duvaud S., Wilkins M.R., Appel R.D., Walker J.M. (2005). The Proteomics Protocols Handbook.

[60] Shaner N.C., Patterson G.H., Davidson M.W. (2007). Advances in fluorescent protein technology. J. Cell Sci..

[61] Li S.-A., Meng X.Y., Zhang Y.J., Chen C.L., Jiao Y.X., Zhu Y.Q. (2024). Progress in pH-Sensitive sensors: essential tools for organelle pH detection, spotlighting mitochondrion and diverse applications. Front. Pharmacol..

[62] Poëa-Guyon S., Pasquier H., Mérola F., Morel N., Erard M. (2013). The enhanced cyan fluorescent protein: a sensitive pH sensor for fluorescence lifetime imaging. Anal. Bioanal. Chem..

[63] Llopis J., McCaffery J.M., Miyawaki A., Farquhar M.G., Tsien R.Y. (1998). Measurement of cytosolic, mitochondrial, and Golgi pH in single living cells with green fluorescent proteins. Proc. Natl. Acad. Sci. U. S. A..

[64] Haupts U., Maiti S., Schwille P., Webb W.W. (1998). Dynamics of fluorescence fluctuations in green fluorescent protein observed by fluorescence correlation spectroscopy. Proc. Natl. Acad. Sci. U. S. A..

[65] Wachter R.M., King B.A., Heim R., Kallio K., Tsien R.Y., Boxer S.G. (1997). Crystal structure and photodynamic behavior of the blue emission variant Y66H/Y145F of green fluorescent protein. Biochemistry.

[66] Ormö M., Cubitt A.B., Kallio K., Gross L.A., Tsien R.Y., Remington S.J. (1996). Crystal structure of the Aequorea victoria green fluorescent protein. Science.

[67] Liao J., Song Y., Liu Y. (2015). A new trend to determine biochemical parameters by quantitative FRET assays. Acta Pharmacol. Sin..

[68] Johnson K.A. (2019). New standards for collecting and fitting steady state kinetic data. Beilstein J. Org. Chem..

[69] Sacco M.D., Hu Y., Gongora M.V., Meilleur F., Kemp M.T., Zhang X. (2022). The P132H mutation in the main protease of Omicron SARS-CoV-2 decreases thermal stability without compromising catalysis or small-molecule drug inhibition. Cell Res..

[70] Zhang J.-H., Chung T.D.Y., Oldenburg K.R.A. (1999). Simple statistical parameter for use in evaluation and validation of high throughput screening assays. J. Biomol. Screen..

[71] Rut W., Groborz K., Zhang L., Sun X., Zmudzinski M., Pawlik B. (2021). SARS-CoV-2 Mpro inhibitors and activity-based probes for patient-sample imaging. Nat. Chem. Biol..

[72] Su H., Yao S., Zhao W., Zhang Y., Liu J., Shao Q. (2021). Identification of pyrogallol as a warhead in design of covalent inhibitors for the SARS-CoV-2 3CL protease. Nat. Commun..

[73] Wang Z., Wang H.-Y., Feng H. (2013). A simple and reproducible method for directed evolution: combination of random mutation with dITP and DNA fragmentation with endonuclease V. Mol. Biotechnol..

[74] MacDonald E.A., Frey G., Namchuk M.N., Harrison S.C., Hinshaw S.M., Windsor I.W. (2021). Recognition of divergent viral substrates by the SARS-CoV-2 main protease. ACS Infect. Dis..

[75] Fan K., Wei P., Feng Q., Chen S., Huang C., Ma L. (2004). Biosynthesis, purification, and substrate specificity of severe acute respiratory syndrome coronavirus 3C-like proteinase. J. Biol. Chem..

[76] Pablos I., Machado Y., de Jesus H.C.R., Mohamud Y., Kappelhoff R., Lindskog C. (2021). Mechanistic insights into COVID-19 by global analysis of the SARS-CoV-2 3CLpro substrate degradome. Cell Rep..

[77] Muramatsu T., Kim Y.T., Nishii W., Terada T., Shirouzu M., Yokoyama S. (2013). Autoprocessing mechanism of severe acute respiratory syndrome coronavirus 3C-like protease (SARS-CoV 3CLpro) from its polyproteins. FEBS J..

[78] Yang H., Yang M., Ding Y., Liu Y., Lou Z., Zhou Z. (2003). The crystal structures of severe acute respiratory syndrome virus main protease and its complex with an inhibitor. Proc. Natl. Acad. Sci. U. S. A..

[79] Lee J., Worrall L.J., Vuckovic M., Rosell F.I., Gentile F., Ton A.T. (2020). Crystallographic structure of wild-type SARS-CoV-2 main protease acyl-enzyme intermediate with physiological C-terminal autoprocessing site. Nat. Commun..

[80] Chen S., Jonas F., Shen C., Hilgenfeld R., Higenfeld R. (2010). Liberation of SARS-CoV main protease from the viral polyprotein: N-terminal autocleavage does not depend on the mature dimerization mode. Protein Cell.

[81] Li C., Qi Y., Teng X., Yang Z., Wei P., Zhang C. (2010). Maturation mechanism of severe acute respiratory syndrome (SARS) coronavirus 3C-like proteinase. J. Biol. Chem..

[82] Wolff G., Limpens R.W.A.L., Zevenhoven-Dobbe J.C., Laugks U., Zheng S., de Jong A.W.M. (2020). A molecular pore spans the double membrane of the coronavirus replication organelle. Science.

[83] Oudshoorn D., Rijs K., Limpens R.W.A.L., Groen K., Koster A.J., Snijder E.J. (2017). Expression and cleavage of Middle East respiratory syndrome coronavirus nsp3-4 polyprotein induce the formation of double-membrane vesicles that mimic those associated with coronaviral RNA replication. mBio.

[84] Gosert R., Kanjanahaluethai A., Egger D., Bienz K., Baker S.C. (2002). RNA replication of mouse hepatitis virus takes place at double-membrane vesicles. J. Virol..

[85] Knoops K., Kikkert M., van den Worm S.H.E., Zevenhoven-Dobbe J.C., van der Meer Y., Koster A.J. (2008). SARS-coronavirus replication is supported by a reticulovesicular network of modified endoplasmic reticulum. PLoS Biol..

[86] Shaqra A.M., Zvornicanin S.N., Huang Q.Y.J., Lockbaum G.J., Knapp M., Tandeske L. (2022). Defining the substrate envelope of SARS-CoV-2 main protease to predict and avoid drug resistance. Nat. Commun..

[87] Xiao Y., Ma Q., Restle T., Shang W., Svergun D.I., Ponnusamy R. (2012). Nonstructural proteins 7 and 8 of feline coronavirus form a 2:1 heterotrimer that exhibits primer-independent RNA polymerase activity. J. Virol..

[88] Kirchdoerfer R.N., Ward A.B. (2019). Structure of the SARS-CoV nsp12 polymerase bound to nsp7 and nsp8 co-factors. Nat. Commun..

[89] Subissi L., Posthuma C.C., Collet A., Zevenhoven-Dobbe J.C., Gorbalenya A.E., Decroly E. (2014). One severe acute respiratory syndrome coronavirus protein complex integrates processive RNA polymerase and exonuclease activities. Proc. Natl. Acad. Sci. U. S. A..

[90] Iketani S., Mohri H., Culbertson B., Hong S.J., Duan Y., Luck M.I. (2022). Multiple pathways for SARS-CoV-2 resistance to nirmatrelvir. Nature.

[91] Kneller D.W., Phillips G., O’Neill H.M., Jedrzejczak R., Stols L., Langan P. (2020). Structural plasticity of SARS-CoV-2 3CL Mpro active site cavity revealed by room temperature X-ray crystallography. Nat. Commun..

[92] Estrada E. (2020). Topological analysis of SARS CoV-2 main protease. Chaos.

[93] Bzówka M., Mitusińska K., Raczyńska A., Samol A., Tuszyński J.A., Góra A. (2020). Structural and evolutionary analysis indicate that the SARS-CoV-2 Mpro is a challenging target for small-molecule inhibitor design. Int. J. Mol. Sci..

[94] Lee J.T., Yang Q., Gribenko A., Perrin B.S., Zhu Y., Cardin R. (2022). Genetic surveillance of SARS-CoV-2 Mpro reveals high sequence and structural conservation prior to the introduction of protease inhibitor paxlovid. mBio.

[95] Singer J., Gifford R., Cotten M., Robertson D. (2020). CoV-GLUE: a web application for tracking SARS-CoV-2 genomic variation. Preprint.

[96] Flynn J.M., Samant N., Schneider-Nachum G., Barkan D.T., Yilmaz N.K., Schiffer C.A. (2022). Comprehensive fitness landscape of SARS-CoV-2 Mpro reveals insights into viral resistance mechanisms. Elife.

[97] Wickham H. (2016).

[98] R Core Team (2022).

[99] Kyte J., Doolittle R.F. (1982). A simple method for displaying the hydropathic character of a protein. J. Mol. Biol..

[100] Krissinel E., Henrick K. (2007). Inference of macromolecular assemblies from crystalline state. J. Mol. Biol..

[101] Sacco M.D., Ma C., Lagarias P., Gao A., Townsend J.A., Meng X. (2020). Structure and inhibition of the SARS-CoV-2 main protease reveal strategy for developing dual inhibitors against Mpro and cathepsin L. Sci. Adv..

[102] Ullrich S., Sasi V.M., Mahawaththa M.C., Ekanayake K.B., Morewood R., George J. (2021). Challenges of short substrate analogues as SARS-CoV-2 main protease inhibitors. Bioorg. Med. Chem. Lett..

[103] Kuo C.-J., Chao T.L., Kao H.C., Tsai Y.M., Liu Y.K., Wang L.H.C. (2021). Kinetic characterization and inhibitor screening for the proteases leading to identification of drugs against SARS-CoV-2. Antimicrob. Agents Chemother..

[104] Costanzi E., Kuzikov M., Esposito F., Albani S., Demitri N., Giabbai B. (2021). Structural and biochemical analysis of the dual inhibition of MG-132 against SARS-CoV-2 main protease (Mpro/3CLpro) and human cathepsin-L. Int. J. Mol. Sci..

[105] Liu C., Boland S., Scholle M.D., Bardiot D., Marchand A., Chaltin P. (2021). Dual inhibition of SARS-CoV-2 and human rhinovirus with protease inhibitors in clinical development. Antivir. Res..

[106] Jumper J., Evans R., Pritzel A., Green T., Figurnov M., Ronneberger O. (2021). Highly accurate protein structure prediction with AlphaFold. Nature.

[107] Evans R., O’Neill M., Pritzel A., Antropova N., Senior A., Green T. (2022). Protein complex prediction with AlphaFold-Multimer. bioRxiv.

[108] Courouble V.V., Dey S.K., Yadav R., Timm J., Harrison J.J.E.K., Ruiz F.X. (2023). Revealing the structural plasticity of SARS-CoV-2 nsp7 and nsp8 using structural proteomics. J. Am. Soc. Mass Spectrom..

[109] Jochheim F.A., Tegunov D., Hillen H.S., Schmitzová J., Kokic G., Dienemann C. (2021). The structure of a dimeric form of SARS-CoV-2 polymerase. Commun Biol.

[110] Wilamowski M., Hammel M., Leite W., Zhang Q., Kim Y., Weiss K.L. (2021). Transient and stabilized complexes of Nsp7, Nsp8, and Nsp12 in SARS-CoV-2 replication. Biophys J..

