# Supplementary material for: Kinetic comparison of all eleven viral polyprotein cleavage site processing events by SARS-CoV-2 main protease using a linked protein FRET platform
Source: J Biol Chem. 2024 May 15;300(6):107367. doi: 10.1016/j.jbc.2024.107367 (PMC11209022; doi:10.1016/j.jbc.2024.107367)
Supplement: Supplemental Figures S1–S9 and Tables S1–S4 [file mmc1.docx]

**Kinetic comparison of all eleven viral polyprotein cleavage site processing events by SARS-CoV-2 main protease using a linked protein FRET platform**

Calem Kenward^1^, Marija Vuckovic^1^, Mark Paetzel^2†^ and Natalie C.J. Strynadka^1†^

**Supporting Information:**

Figure S1. Sequence conservation of M^pro^ polyprotein cut sites across human pathogenic *coronaviridae* viruses

Figure S2. Representative purification of ECFP-C*x*-EYFP substrates

Figure S3. MALDI-TOF mass spectrometry of ECFP-C*x*-EYFP substrates

Figure S4. Cleavage of ECFP-C*x*-EYFP substrates by M^pro^

Figure S5. Figure S5. Processing of ECFP-Cx-EYFP substrate constructs by Mpro restores fluorescent properties of ECFP and EYFP products

Figure S6. Cleavage of ECFP-C4-EYFP results in increase in ECFP emission and proportional decrease in EYFP response.

Figure S7. Screening effect of M^pro^ mutations on proteolytic activity and inhibitor activity using ECFP-C15-EYFP system.

Figure S8. Thermostability average of M^pro^ cut site sequences.

Figure S9. Binding efficiency of polyprotein cut site sequences within active site of Mpro are determined by sequence properties.

Table S1. M^pro^ polyprotein cut site sequences in human pathogenic *Coronaviridae*

Table S2. Previous peptide FRET substrates and associated reactivity

Table S3: Optimized construct, gene, and primer sequences used in this study

Table S4. Polyprotein cut site sequence biding in published structures

^1^Department of Biochemistry and Molecular Biology and Centre for Blood Research, The University of British Columbia, Vancouver, British Columbia, Canada; ^2^Department of Molecular Biology and Biochemistry, Simon Fraser University, Burnaby, British Columbia, Canada

† Correspondence to [mpaetzel@sfu.ca](mailto:mpaetzel@sfu.ca) and [ncjs@mail.ubc.ca](mailto:ncjs@mail.ubc.ca)


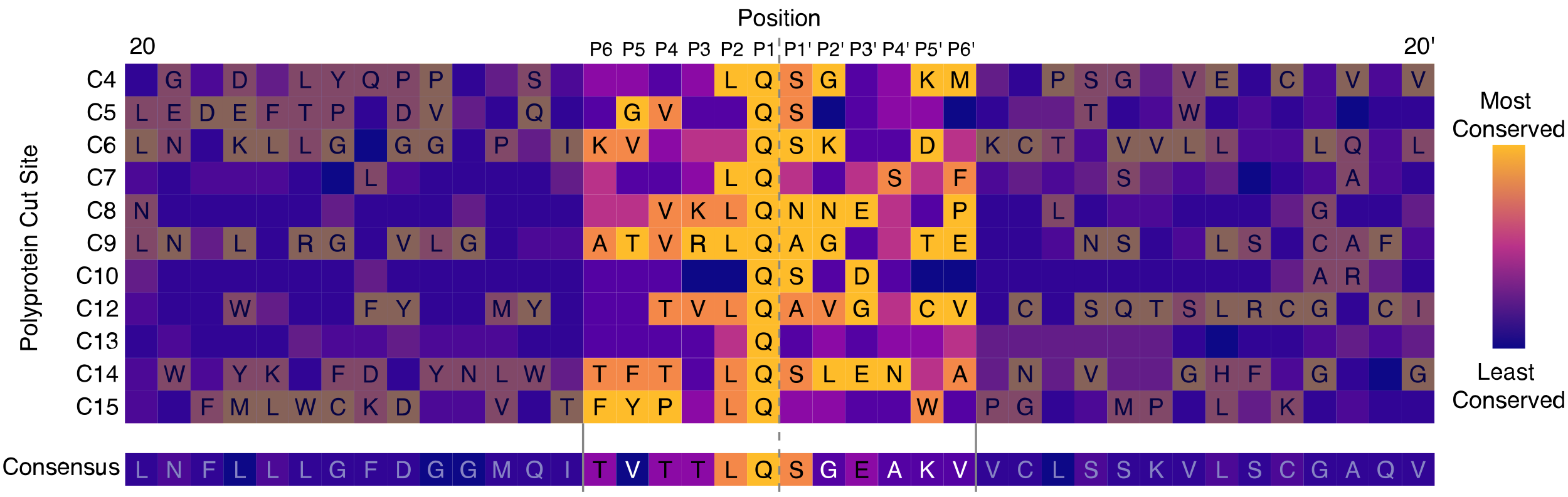


**Figure S1. Sequence conservation of M^pro^ polyprotein cut sites across human pathogenic *coronaviridae* viruses**

Sequence alignment of each polyprotein cut site sequence among seven commonly studied human pathogenic coronaviruses: SARS-CoV-2, SARS-CoV-1, MERS, HCov-HKU1, HCoV-OC4, HCoV-NL63, and HCoV-229E. The sequences analysed with associated Uniprot accession IDs are summarized in Table S1. Each cut site sequence is aligned on the conserved glutamate that demarks the P1 position. The six flanking residues on either side of this junction (N-terminal: P6-P1; C-terminal: P1’-P6’; following Schechter–Berger nomenclature) that are known to directly interact with the binding groove of M^pro^ are highlighted. Higher residue maintenance at each position is indicated by brighter colouring, with invariant residues shown in bright orange. For residues with greater than 50% conservation (present in ≥4/7 species), the residue is indicated in black. Consensus sequence across all seven human pathogenic coronaviruses and all cut sites (see Table S1) is shown below, with equal colouring scheme but with most commonly observed residue at each position shown in black or white for clarity.

**Figure S2. Representative purification of ECFP-C*x*-EYFP substrates**

(A) 15% SDS-PAGE gel with samples taken at various point of the purification process starting with lysis (Ly) through Ni^2+^ immobilized metal ion chromatography (IMAC) flowthrough (FT), wash (W) and Elution (E) and subsequent cleavage (C) with thrombin protease to remove the His-tag. Following another round of Ni^2+^-IMAC, instead collecting the flowthrough containing the cleaved substrate and discarding the elution of uncleaved protein. The final purification step was size-exclusion chromatography using a Superdex 200 (SD200) column. All samples stained with stained with coomassie brilliant blue R. Collected fractions A5, A6, and A7 boxed in red correspond to the peak boxed in red in the chromatogram in panel (B).

**Figure S3. MALDI-TOF mass spectrometry of ECFP-C*x*-EYFP substrates**

Fluorescent substrates were purified to isolation using Ni^2+^-IMAC and SEC (See Figure S1) and the identity and purity of three representative constructs, C4 (A), C6 (B), and C15 (C) were verified using mass spectrometry. Peaks at ~66 kDa correspond to BSA which was used as an internal calibrant to achieve an improved mass accuracy. (D) Expected molecular weight of construct before (uncut) and after thrombin protease cleavage (cut) to remove hexa-histidine expression tag shown for each construct, with measured m/z values shown for substrates evaluated, in each case showing a calculated mass close to expected value of cut protein.

**Figure S4. Cleavage of ECFP-Cx-EYFP substrates by M^pro^**

(A) ECFP-C*x*-EYFP constructs (10 μM) prior to (B) incubation overnight with 100 nM M^pro^ (see Figure 3.3. for corresponding emission wave scans) showing a clear shift from ~60 kDa to ~30 kDa after incubation with M^pro^. C6, C8, and C10 all show a remaining upper band at 60 kDa after incubation with M^pro^ corresponding to uncleaved substrate.

**Figure S5. Processing of ECFP-Cx-EYFP substrate constructs by M^pro^ restores fluorescent properties of ECFP and EYFP products**

(A) Cleavage of 50 μM ECFP-C4-EYFP into free ECFP and EYFP by 1 μM M^pro^ at room temperature. Samples were taken at regular intervals and mixed with SDS loading dye to stop the reaction prior to loading onto a 15% SDS-PAGE gel and staining with Coomassie blue. (B) Gel densitometry of ECFP-C4-EYFP, ECFP, and EYFP bands identified in (A), with error bars indicating standard deviation. The similar molecular weight of ECFP and EYFP halves of the cleaved substrate results in significant overlap, making deconvolution of EYFP and ECFP bands difficult. (C) Clear shift of emission wavelength from EYFP maximum at 528 nm to 477 nm ECFP emission is observed as the reaction progresses (black / grey to blue). (D) Emission wave scan of free ECFP (cyan) or free EYFP (yellow) after excitation with 434 nm or 477 nm light, respectively. Both ECFP and EYFP fluoresce at the emission maximum of EYFP (~528nm), with the emission of ECFP being ~1/3^rd^ of EYFP as shown. (E) Fluorescence of ECFP and EYFP measured at their emission maxima over the course of CLY2 processing by M^pro^. (F) Comparison of ECFP-C4-EYFP processing by M^pro^ as monitored by gel (from A; purple line) and fluorescence assays (from E; blue and yellow lines for ECFP and EYFP respectively). All values normalized to maximum and minimum values observed in respective experiments to allow for direct comparison.

**Figure S6. Cleavage of ECFP-C4-EYFP results in increase in ECFP emission and proportional decrease in EYFP response.**

ECFP-C4-EYFP (50 μM) cleavage by 50 nM of M^pro^ at room temperature was monitored via fluorescence at 477 nm (A; ECFP) and 528 nm (B; EYFP). Measurements were taken every 80 seconds for 240 minutes after excitation at 434 nm. Initial velocity was determined from the linear portion of each curve, indicated by dotted lines at 20 minutes. A zoomed-in view of the first 20 minutes of cleavage showed measurements taken every 40 seconds, converted to ECFP (C) and EYFP (D) concentrations using standard curves (see Figure 3.5). Similar plots were generated for each replicate reaction between ECFP-Cx-EYFP substrates and M^pro^ (summarized in Figure 3.5). (E) Cleavage of 50 μM ECFP-C4-EYFP into free ECFP and EYFP by 50 nM M^pro^ at room temperature. Samples were taken at regular intervals and mixed with SDS loading dye to stop the reaction prior to loading onto a 15% SDS-PAGE gel and staining with Coomassie blue. (F) Gel densitometry of ECFP-C4-EYFP, ECFP, and EYFP bands identified in (E), with error bars indicating standard deviation.

**Figure S7. Screening effect of M^pro^ mutations on proteolytic activity and inhibitor activity using ECFP-C15-EYFP system.**

(A) Response curve of 10 μM ECFP-C15-EYFP substrate with 100 nM M^pro^ WT (filled circles) or M^pro^ P132H (squares) and nirmatrelvir concentrations ranging from 1 nM to1 μM. Substrate concentration matched to ECFP-C4-EYFP in Figure 6B, however the lower reactivity of C15 required increasing protease concentration as C15 is significantly less reactive than C4. Calculated IC_50_ from each dose curve shows that while nirmatrelvir is still a potent inhibitor in either case, there is a clear difference in inhibitory effect between WT and P132H M^pro^ variants. Each point represents mean of n=3 runs collected over 20 minutes, with error bars showing ±1 standard deviation. (B) ECFP-C4-EYFP cleavage dose curve of a non-peptidomimetic direct-acting SARS-CoV-2 antiviral, C5a. Calculated IC_50_ of 2.10 μM closely match reported literature value of 4.5 ± 1.0 μM^1^.

**Figure S8. Thermostability average of M^pro^** **cut site sequences.**

Aggregation temperature of each ECFP-C*x*-EYFP substrate was determined using differential light scattering. The buffer conditions were the same as those used in the kinetic assays (20 mM HEPES pH 7.5, 150 mM NaCl, 1mM DTT, 1mM EDTA). Mineral oil (10 μL) was pipetted on top of 10 μL of 2.5 μM to 40 μM substrate in a 384 well plate. Each concentration of substrate was loaded into five separate wells as replicates. Shown is the mean calculated aggregation temperature (T_agg_) of three repeat experiments across all substrate concentrations, with error bars showing ±1 standard deviation (n=15), higher (red) values indicating higher thermostability.

**Figure S9. Binding efficiency of polyprotein cut site sequences within active site of Mpro are determined by sequence properties.**

Subset of structures of polyprotein cut site sequences bound within the active site of Mpro highlight key differences in binding. (A) Comparing structure of sequences with relatively small (C4 - PDB ID: 7MB4^2^, C6 - PDB ID: 7MB6^2^) versus large (C10 - PDB ID: 7MB9^2^, C15 - PDB ID: 7TC6^2^) side chain steric bulk. Of these sequences, C4 and C15 cleave much more efficiently than C6 and C10 which are among the lest reactive, as summarized by Figure 5B. Structural properties of each polyprotein cut site specificity sequence (P6-P1 only) summarized as mean of (B) accessible surface area of unbound residues, (C) buried surface area of residues in interface, (D) average B-factor, and (E) calculated grand average of hydropathicity (GRAVY).^3^ Colors in each plot are proportional to corresponding value in each plot to better highlight differences. Structures analyzed with associated values are detailed in Table S4. Accessible surface area and buried surface area were calculated using ePISA^4^ and residue average B-factors were averaged across available structures.

| **Table S1. M^pro^ polyprotein cut site sequences in human pathogenic *Coronaviridae*** | | | | |
| --- | --- | --- | --- | --- |
| Cut Site | Region | | P1\|P1’ Position | Sequence (P6-P6’ bolded) |
| **SARS-CoV-2** (Uniprot ID: P0DTD1) | | | |  |
| **C4** | nsp4 / nsp5 | | 3263-3264 | SGSDVLYQPPQTSI**TSAVLQ\|SGFRKM**AFPSGKVEGCMVQV |
| **C5** | nsp5 / nsp6 | | 3569-3570 | LEDEFTPFDVVRQC**SGVTFQ\|SAVKRT**IKGTHHWLLLTILT |
| **C6** | nsp6 / nsp7 | | 3859-3860 | LNIKLLGVGGKPCI**KVATVQ\|SKMSDV**KCTSVVLLSVLQQL |
| **C7** | nsp7 / nsp8 | | 3942-3943 | GAVDINKLCEEMLD**NRATLQ\|AIASEF**SSLPSYAAFATAQE |
| **C8** | nsp8 / nsp9 | | 4140-4141 | NLAWPLIVTALRAN**SAVKLQ\|NNELSP**VALRQMSCAAGTTQ |
| **C9** | nsp9 / nsp10 | | 4253-4254 | LNNLNRGMVLGSLA**ATVRLQ\|AGNATE**VPANSTVLSFCAFA |
| **C10** | nsp10 / nsp11 | | 4392-4393 | CGMWKGYGCSCDQL**REPMLQ\|SADAQS**FLNRVCGVSAARLT |
| **C12** | nsp12 / nsp13 | | 5324-5325 | SRYWEPEFYEAMYT**PHTVLQ\|AVGACV**LCNSQTSLRCGACI |
| **C13** | nsp13 / nsp14 | | 5925-5926 | YDKLQFTSLEIPRR**NVATLQ\|AENVTG**LFKDCSKVITGLHP |
| **C14** | nsp14 / nsp15 | | 6452-6453 | LWVYKQFDTYNLWN**TFTRLQ\|SLENVA**FNVVNKGHFDGQQG |
| **C15** | nsp15 / nsp16 | | 6798-6799 | ISFMLWCKDGHVET**FYPKLQ\|SSQAWQ**PGVAMPNLYKMQRM |
|  |  | |  |  |
| **SARS-CoV-1** (Uniprot ID: P0C6X7) | | | |  |
| **C4** | nsp4 / nsp5 | | 3240-3241 | SGADVLYQPPQTSI**TSAVLQ\|SGFRKM**AFPSGKVEGCMVQV |
| **C5** | nsp5 / nsp6 | | 3546-3547 | LEDEFTPFDVVRQC**SGVTFQ\|GKFKKI**VKGTHHWMLLTFLT |
| **C6** | nsp6 / nsp7 | | 3836-3837 | LNIKLLGIGGKPCI**KVATVQ\|SKMSDV**KCTSVVLLSVLQQL |
| **C7** | nsp7 / nsp8 | | 3919-3920 | GAVDINRLCEEMLD**NRATLQ\|AIASEF**SSLPSYAAYATAQE |
| **C8** | nsp8 / nsp9 | | 4117-4118 | NLAWPLIVTALRAN**SAVKLQ\|NNELSP**VALRQMSCAAGTTQ |
| **C9** | nsp9 / nsp10 | | 4230-4231 | LNNLNRGMVLGSLA**ATVRLQ\|AGNATE**VPANSTVLSFCAFA |
| **C10** | nsp10 / nsp11 | | 4369-4370 | CGMWKGYGCSCDQL**REPLMQ\|SADAST**FLNRVCGVSAARLT |
| **C12** | nsp12 / nsp13 | | 5301-5302 | SRYWEPEFYEAMYT**PHTVLQ\|AVGACV**LCNSQTSLRCGACI |
| **C13** | nsp13 / nsp14 | | 5902-5903 | YDKLQFTSLEIPRR**NVATLQ\|AENVTG**LFKDCSKIITGLHP |
| **C14** | nsp14 / nsp15 | | 6429-6430 | LWIYKQFDTYNLWN**TFTRLQ\|SLENVA**YNVVNKGHFDGHAG |
| **C15** | nsp15 / nsp16 | | 6775-6776 | ISFMLWCKDGHVET**FYPKLQ\|ASQAWQ**PGVAMPNLYKMQRM |
|  |  | |  |  |
| **MERS** (Uniprot ID: K9N7C7) | | | |  |
| **C4** | nsp4 / nsp5 | | 3247-3248 | TGSDLLYQPPNCSI**TSGVLQ\|SGLVKM**SHPSGDVEACMVQV |
| **C5** | nsp5 / nsp6 | | 3553-3554 | LEDEFTPEDVNMQI**MGVVMQ\|SGVRKV**TYGTAHWLFATLVS |
| **C6** | nsp6 / nsp7 | | 3845-3846 | LNFKLIGIGGTPCI**KVAAMQ\|SKLTDL**KCTSVVLLSVLQQL |
| **C7** | nsp7 / nsp8 | | 3928-3929 | GNVDLDALASDIFD**TPSVLQ\|ATLSEF**SHLATFAELEAAQK |
| **C8** | nsp8 / nsp9 | | 4127-4128 | LTWPLVLECTRAST**SAVKLQ\|NNEIKP**SGLKTMVVSAGQEQ |
| **C9** | nsp9 / nsp10 | | 4237-4238 | LNNLHRGQVLGHIA**ATVRLQ\|AGSNTE**FASNSSVLSLVNFT |
| **C10** | nsp10 / nsp11 | | 4377-4378 | QYWIGYGCNCDSLR**QAALPQ\|SKDSNF**LKRVRGSIVNARIE |
| **C12** | nsp12 / nsp13 | | 5310-5311 | AKFWEEAFYRDLYS**SPTTLQ\|AVGSCV**VCHSQTSLRCGTCI |
| **C13** | nsp13 / nsp14 | | 5908-5909 | QALFESLEFTELSF**TNYKLQ\|SQIVTG**LFKDCSRETSGLSP |
| **C14** | nsp14 / nsp15 | | 6432-6433 | LWCYKTFDIYNLWS**TFTKVQ\|GLENIA**FNFVKQGHFIGVEG |
| **C15** | nsp15 / nsp16 | | 6775-6776 | IEFMLWCKDGQVQT**FYPRLQ\|ASADWK**PGHAMPSLFKVQNV |
|  |  | |  |  |
| **HCov-HKU1** (Uniprot ID: P0C6X2) | | | |  |
| **C4** | nsp4 / nsp5 | | 3334-3335 | NGNDVLYQPPTASV**STSFLQ**\|**SGIVKM**VSPTSKIEPCIVSV |
| **C5** | nsp5 / nsp6 | | 3637-3638 | FEDELAPSDVYQQL**AGVKLQ**\|**SKTKRF**IKETIYWILISTFL |
| **C6** | nsp6 / nsp7 | | 3924-3925 | LNLKLLGIGGVPVI**EVSQIQ\|SKLTDV**KCANVVLLNCLQHL |
| **C7** | nsp7 / nsp8 | | 4016-4017 | SIDEVSDDYVQDST**VLQALQ\|SEFVNM**ASFVEYEVAKKNLA |
| **C8** | nsp8 / nsp9 | | 4210-4211 | NWPLVIIANRYNEV**ANAVMQ\|NNELMP**HKLKIQVVNSGSDM |
| **C9** | nsp9 / nsp10 | | 4320-4321 | CNTLARGWVVGTLS**STIRLQ\|AGVATE**YAANSSILSLCAFS |
| **C10** | nsp10 / nsp11 | | 4457-4458 | VCGFWRDGSCSCVG**SSVAVQ\|SKDLNF**LNRVRGTSVNARLV |
| **C12** | nsp12 / nsp13 | | 5385-5386 | LKFTEESFYKNMYL**KSAVMQ\|SVGACV**VCSSQTSLRCGSCI |
| **C13**^‡^ | nsp13 / nsp14 | | ‡ | NMQLFESLNFITL**PLDKIQ\|QTLPRL**HCTTNLFKDCSKSC |
| **C14** | nsp14 / nsp15 | | 6509-6510 | FWVYKNFDFYNLWN**TFTTLQ\|SLENVI**YNLVNVGHYDGRTG |
| **C15** | nsp15 / nsp16 | | 6883-6884 | FQFMLWCNDNKIMT**FYPKMQ\|ATNDWK**PGYSMPVLYKYLNV |
| ‡ Non-specific or ambiguous | | | | |
| Table S1. M^pro^ polyprotein cut site sequences in human pathogenic *Coronaviridae* (continued) | | | | |
| Cut Site | | Region | P1\|P1’ Position | Sequence |
| **HCoV-OC4** (Uniprot ID: P0C6X6) | | | |  |
| **C4** | | nsp4 / nsp5 | 3246-3247 | NGSDVLYQPPTASV**STSFLQ\|SGIVKM**VNPTSKVEPCVVSV |
| **C5** | | nsp5 / nsp6 | 3549-3550 | FEDELTPSDVYQQL**AGIKLQ\|SKRTRL**FKGTVCWIMASTFL |
| **C6** | | nsp6 / nsp7 | 3836-3837 | LNFKLLGIGGVPII**EVSQFQ\|SKLTDV**KCANVVLLNCLQHL |
| **C7** | | nsp7 / nsp8 | 3925-3926 | CLTSIEEVCDDYAK**DNTVLQ\|ALQSEF**VNMASFVEYEVAKK |
| **C8** | | nsp8 / nsp9 | 4122-4123 | NWPLVIIANRYNEV**SATVLQ\|NNELMP**AKLKIQVVNSGPDQ |
| **C9** | | nsp9 / nsp10 | 4232-4233 | CNTLARGWVVGTIS**STVRLQ\|AGTATE**YASNSSILSLCAFS |
| **C10** | | nsp10 / nsp11 | 4369-4370 | VCGFWRDGSCSCVS**TDTTVQ\|SKDTNF**LNRVRGASVDARLV |
| **C12** | | nsp12 / nsp13 | 5297-5298 | QKFTDESFYKNMYL**RSAVMQ\|SVGACV**VCSSQTSLRCGSCI |
| **C13** | | nsp13 / nsp14 | 5900-5901 | LQFTTLTLDKVPQA**VETKVQ\|CSTNLF**KDCSKSYSGYHPAH |
| **C14** | | nsp14 / nsp15 | 6421-6422 | FWVYKTFDFYNLWN**TFTKLQ\|SLENVV**YNLVKTGHYTGQAG |
| **C15** | | nsp15 / nsp16 | 6796-6797 | FQFMLWCNDEKVMT**FYPRLQ\|AASDWK**PGYSMPVLYKYLNS |
|  | |  |  |  |
| **HCoV-NL63** (Uniprot ID: P0C6X5) | | | |  |
| **C4** | | nsp4 / nsp5 | 2939-2940 | KDHNDMLYSPPTIS**YNSTLQ\|SGLKKM**AQPSGCVERCVVRV |
| **C5** | | nsp5 / nsp6 | 3242-3243 | LCDEFTLAEVVKQM**YGVNLQ\|SGKVIF**GLKTMFLFSVFFTM |
| **C6** | | nsp6 / nsp7 | 3521-3522 | LSFKLLGIGGDRCI**KISTVQ\|SKLTDL**KCTNVVLLGCLSSM |
| **C7** | | nsp7 / nsp8 | 3604-3605 | SDFGLDGLIDSYFD**NSSTLQ\|SVASSF**VSMPSYIAYENARQ |
| **C8** | | nsp8 / nsp9 | 3799-3800 | NVETLTWPLILNCE**RVVKLQ\|NNEIMP**GKLKQKPMKAEGDG |
| **C9** | | nsp9 / nsp10 | 3908-3909 | LNTLRRGAVLGFIG**ATIRLQ\|AGKQTE**LAVNSGLLTACAFS |
| **C10** | | nsp10 / nsp11 | 4043-4044 | CNVCGCWLGHGCAC**DRTTIQ\|SVDISY**LNRARGSSAARLEP |
| **C12** | | nsp12 / nsp13 | 4970-4971 | DKFWCEDFYASMYE**NSTILQ\|AAGLCV**VCGSQTVLRCGDCL |
| **C13**^‡^ | | nsp13 / nsp14 | ‡ | TLFDSLKFFEIKH**ADLHSSQ\|VCGLFK**NCTRTPLNLPPTHA |
| **C14** | | nsp14 / nsp15 | 6085-6086 | VPHSFDVYNLWQIF**IETNLQ\|SLENIA**FNVVKKGCFTGVDG |
| **C15** | | nsp15 / nsp16 | 6429-6430 | YRWMLWCKDNHLST**FYPQLQ\|SAEWKC**GYAMPQIYKLQRMC |
|  | |  |  |  |
| **HCoV-229E** (Uniprot ID: P0C6X1) | | | |  |
| **C4** | | nsp4 / nsp5 | 2965-2966 | RDHNDILYTPPTVS**YGSTLQ\|AGLRKM**AQPSGFVEKCVVRV |
| **C5** | | nsp5 / nsp6 | 3267-3268 | LNDEFSINEVVKQM**FGVNLQ\|SGKTTS**MFKSISLFAGFFVM |
| **C6** | | nsp6 / nsp7 | 3546-3547 | LSFKLMGIGGPRTI**KVSTVQ\|SKLTDL**KCTNVVLMGILSNM |
| **C7** | | nsp7 / nsp8 | 3629-3630 | SDFGLGDLVDSYFE**NDSILQ\|SVASSF**VGMPSFVAYETARQ |
| **C8** | | nsp8 / nsp9 | 3824-3825 | NQEILVWPLILTCE**RVVKLQ\|NNEIMP**GKMKVKATKGEGDG |
| **C9** | | nsp9 / nsp10 | 3933-3934 | LNNLRRGAVLGYIG**ATVRLQ\|AGKQTE**FVSNSHLLTHCSFA |
| **C10** | | nsp10 / nsp11 | 4068-4069 | CKVCGCWLNHGCTC**DRTAIQ\|SFDNSY**LNRVRGSSAARLEP |
| **C12** | | nsp12 / nsp13 | 4995-4996 | SKFWDESFYASMYE**KSTVLQ\|AAGLCV**VCGSQTVLRCGDCL |
| **C13** | | nsp13 / nsp14 | 5592-5593 | SDRTLFDALKFFEI**TMTDLQ\|SESSCG**LFKDCARNPIDLPP |
| **C14** | | nsp14 / nsp15 | 6110-6111 | VPTTFDCYNLWQTF**TEVNLQ\|GLENIA**FNVVNKGSFVGADG |
| **C15** | | nsp15 / nsp16 | 6458-6459 | WRWMLWCKDNAVAT**FYPQLQ\|SAEWKC**GYSMPGIYKTQRMC |
| ‡ Non-specific or ambiguous | | | | |

| **Table S2. Previous peptide FRET substrates and associated reactivity** | | |  |
| --- | --- | --- | --- |
| Substrate | Sequence | k_cat_/K_M_ (M^−1^ s^−1^) | Source |
| nsp4–5-MCA | [MCA]-AVLQ↓SGFR-[K(DNP)]K-NH2 | 14190 ± 420 | (Legare et al., 2021)^5^ |
| nsp4–5-EDANS | [DABCYL]-KTSAVLQ↓SGFRKM-[E(EDANS)]-NH2 | 1960 ± 190 |  |
| nsp4–5-FAM | [DABCYL]-KTSAVLQ↓SGFR-[K(FAM)]K-NH2 | 2448 ± 85 |  |
| nsp5–6-FAM | [DABCYL]-KSGVTFQ↓SAVK-[K(FAM)]K-NH2 | 77.5 ± 4.2 |  |
| nsp6–7-FAM | [DABCYL]-KKVATVQ↓SKMS-[K(FAM)]K-NH2 | 68 ± 10 |  |
| nsp8–9-FAM | [DABCYL]-KSAVKLQ↓NNEL-[K(FAM)]K-NH2 | 6.01 ± 0.61 |  |
| nsp10–12-FAM | [DABCYL]-KREPMLQ↓SADA-[K(FAM)]K-NH2 | 4.74 ± 0.48 |  |
| nsp14–15-FAM | [DABCYL]-KTFTRLQ↓SLEN-[K(FAM)]K-NH2 | 38.0 ± 5.2 |  |
|  |  |  |  |
| 1 (nsp4-nsp5) | 2-Abz-SAVLQ↓SG-Tyr(3-NO_2_)-R-OH | 248.0 ± 89.2 | (Dražić et al., 2021)^6^ |
| 2 | 2-Abz-SVTLQ↓SG-Tyr(3-NO_2_)-R-OH | 212.9 ± 45.5 |  |
| 3 | 2-Abz-VVTLQ↓SG-Tyr(3-NO_2_)-R-OH | 370.4 ± 82.6 |  |
| 4 | 2-Abz-VVTLQ↓SG-[Dap(Dnp)]-R-OH | 229.4 ± 44.2 |  |
|  |  |  |  |
| (nsp4-nsp5) | [DABCYL]-KTSAVLQ↓SGFRKME-[EDANS]-NH2 | 5800 | (Breidenbach et al., 2021)^7^ |
| Boc-Abu-Tle-Leu-Gln-AMC | Boc-Abu-Tle-LQ↓AMC | 604 |  |
|  |  |  |  |
| QS1 | ACC-Gly-Abu-Tle-LQ↓SGFR-Lys(dnp)-Lys-NH2 | 859 ± 57 | (Rut et al., 2021)^8^ |
| QS4 | ACC-Gly-Thz-Tle-LQ↓SGFR-Lys(dnp)Lys-NH2 | 760 ± 50 |  |
| QS3 | ACC-Gly-VKLQ↓SGFR-Gly-Lys(dnp)-LysNH2 | 219 ± 3 |  |
|  |  |  |  |
| nsp4/5 | [DABCYL]-KTSAVLQ↓SGFRKME-[EDANS] | 13000 ± 3000 | (MacDonald et al., 2021)^9^ |
| nsp8/9 | [DABCYL]-KRVVKLQ↓NNELMPE-[EDANS] | 360 ± 70 |  |
| nsp8/9 N1′A | [DABCYL]-KRVVKLQ↓ANELMPE-[EDANS] | 1000 ± 100 |  |
| nsp8/9 N2′A | [DABCYL]-KRVVKLQ↓NAELMPE-[EDANS] | 750 ± 80 |  |
| nsp8/9 N2′D | [DABCYL]-KRVVKLQ↓NDELMPE-[EDANS] | 160 ± 120 |  |
|  |  |  |  |
| (nsp4-nsp5) | [DABCYL]-KTSAVLQ↓SGFRKME-[EDANS] | N/A (K_M_ = 75.41 μM) | (Zhu et al., 2020)^10^ |
| (nsp4-nsp5) | [DABCYL]-KTSAVLQ↓SGFRKME-[EDANS] | 6689 / 5748 | (Sacco et al., 2020)^11^ |
| (nsp4-nsp5) | [DABCYL]-KTSAVLQ↓SGFRKME-[EDANS] | 23 500 | (Ullrich et al., 2021)^12^ |
| (nsp4-nsp5) | [DABCYL]-KTSAVLQ↓SGFRKME-[EDANS] | 5,624 | (Ma et al., 2020)^13^ |
| (nsp4-nsp5) | [DABCYL]-KTSAVLQ↓SGFRKME-[EDANS] | 209 523 ? | (Kuo et al., 2021)^14^ |
| (nsp4-nsp5) | [MCA]-AVLQ↓SGFR-Lys[Dnp]-Lys-NH_2_ | N/A | (Dai et al. 2020)^15^ |
| (nsp4-nsp5) | [MCA]-AVLQ↓SGFR-Lys[Dnp]-Lys-NH_2_ | 28 500 | (Jin et al., 2020)^16^ |
| (nsp4-nsp5) | [MCA]-AVLQ↓SGFR-Lys[Dnp]-Lys-NH_2_ | 25 600 | (Li et al., 2020)^17^ |
|  |  |  |  |
| (nsp4-nsp5) | [DABCYL]-KTSAVLQ↓SGFRKME-[EDANS] | N/A (K_M_ = 18.49 μM) | (Costanzi et. al., 2021)^18^ |
| (nsp4-nsp5) | Ac-TSAVLQSGFRKK-(biotin)-NH2 | 98 333 (from SAMDI-MS) | (Liu et al., 2021)^19^ |
|  |  |  |  |
| F-nsp4-5-Q | H-C(HiLyte™ 488)-TSAVLQ↓SGFRK-(QXL®520)-NH2 | 1766.7 ± 83.3 | (Krichel et al. 2020)^20^ |
| F-nsp7-8-Q | H-C(HiLyte™ 488)-NRATLQ↓AIASK-(QXL®520)-NH2 | 300.0 ± 50.0 | *SARS-CoV-1 |
| F-nsp8-9-Q | H-C(HiLyte™ 488)-SAVKLQ↓NNELK-(QXL®520)-NH2 | 1.7 ± 0.8 | (values converted) |
| F-nsp9-10-Q | H-C(HiLyte™ 488)-ATVRLQ↓AGNAK-(QXL®520)-NH2 | 916.7 ± 33.3 |  |

| **Table S3: Optimized construct, gene, and primer sequences used in this study** | |
| --- | --- |
| The pET28 SUMO-Mpro plasmid expresses His_6_-SUMO-Mpro fusion protein  His_6_-tag  **SUMO**  M^pro^ | -110 -100 -90 -80 -70 -60 -50  **MGSSHHHHHH GSGLVPRGSA SMSDSEVNQE AKPEVKPEVK PETHINLKVS DGSSEIFFKI KKTTPLRRLM**  -40 -30 -20 -10 -1 1 10 20  **EAFAKRQGKE MDSLRFLYDG IRIQADQTPE DLDMEDNDII EAHREQIGG-** SGFRKMAFPS GKVEGCMVQV  30 40 50 60 70 80 90  TCGTTTLNGL WLDDVVYCPR HVICTSEDML NPNYEDLLIR KSNHNFLVQA GNVQLRVIGH SMQNCVLKLK  100 110 120 130 140 150 160  VDTANPKTPK YKFVRIQPGQ TFSVLACYNG SPSGVYQCAM RPNFTIKGSF LNGSCGSVGF NIDYDCVSFC  170 180 190 200 210 220 230  YMHHMELPTG VHAGTDLEGN FYGPFVDRQT AQAAGTDTTI TVNVLAWLYA AVINGDRWFL NRFTTTLNDF  240 250 260 270 280 290 300  NLVAMKYNYE PLTQDHVDIL GPLSAQTGIA VLDMCASLKE LLQNGMNGRT ILGSALLEDE FTPFDVVRQC  306  SGVTFQ |
| Codon optimized M^pro^ gene sequence including N-terminal His_6_-SUMO-tag | 5’‑ ATGGGCAGCAGCCATCACCACCATCATCACGGCTCTGGATTGGTCCCCCGCGGCAGTGCTTCGATGAGCGACTCTGAAGTTAATCAGGAGGCCAAACCTGAGGTAAAACCGGAAGTGAAGCCAGAGACTCATATTAACTTGAAGGTATCCGATGGCTCCTCCGAGATTTTTTTCAAAATTAAAAAAACGACACCTCTGCGCCGTCTTATGGAGGCTTTCGCTAAACGTCAAGGAAAGGAAATGGACTCTCTGCGCTTTTTATATGATGGTATTCGTATTCAGGCCGATCAGACACCGGAAGACCTTGACATGGAAGATAACGACATCATTGAGGCCCACCGTGAACAGATCGGTGGGTCTGGCTTTCGTAAGATGGCCTTCCCATCAGGTAAAGTTGAGGGATGCATGGTGCAGGTTACATGCGGCACTACGACGCTTAACGGCCTGTGGCTCGACGATGTGGTTTATTGCCCACGTCATGTGATTTGCACTTCTGAAGACATGCTGAACCCAAATTATGAAGATTTACTGATTCGCAAAAGTAATCATAATTTTCTGGTACAGGCGGGGAACGTTCAACTGCGCGTCATCGGGCACTCTATGCAGAATTGCGTCCTGAAGCTGAAAGTTGATACTGCGAACCCAAAAACACCAAAATATAAGTTTGTGCGCATTCAACCGGGCCAAACTTTCAGTGTTTTGGCTTGTTATAACGGCAGTCCGTCGGGTGTATATCAGTGCGCAATGCGTCCTAATTTCACGATTAAGGGGTCTTTTCTCAATGGGTCCTGTGGTTCCGTTGGTTTTAATATTGACTATGATTGCGTGTCATTCTGCTATATGCACCATATGGAGTTACCGACCGGAGTGCATGCCGGCACGGATCTGGAGGGCAATTTTTATGGCCCTTTTGTAGATCGTCAGACCGCCCAAGCCGCTGGTACGGATACCACCATCACCGTGAATGTTTTAGCGTGGCTGTACGCAGCGGTGATCAACGGCGACCGTTGGTTTTTGAATCGCTTTACTACAACGTTAAACGATTTCAACCTCGTTGCCATGAAGTACAATTATGAACCCCTCACTCAGGATCACGTCGACATCCTGGGTCCACTGTCGGCGCAGACAGGGATTGCCGTCCTGGATATGTGTGCGTCACTGAAAGAACTGTTGCAAAACGGGATGAACGGCCGTACAATCCTGGGTAGTGCGCTGCTGGAGGATGAGTTTACGCCGTTCGACGTGGTCCGGCAATGTAGTGGCGTGACCTTCCAATAA ‑3’ |
|  |  |
| Modified pET28-CLY2 plasmid expresses His_6_-Th-ECFP-(linker)-EYFP fusion protein  His_6_-tag  Thrombin Cut Site (￬)  ECFP  **GGSGGS rpt**  Mpro cut site (XXX)  EYFP | 10 ￬20 30 40 50 60 70  **MGSSHHHHHH** **SSGLVPRGSH** MVSKGEELFT GVVPILVELD GDVNGHKFSV SGEGEGDATY GKLTLKFICT  80 90 100 110 120 130 140  TGKLPVPWPT LVTTLTWGVQ CFSRYPDHMK QHDFFKSAMP EGYVQERTIF FKDDGNYKTR AEVKFEGDTL  150 160 170 180 190 200 210  VNRIELKGID FKEDGNILGH KLEYNYISHN VYITADKQKN GIKANFKIRH NIEDGSVQLA DHYQQNTPIG  220 230 240 250 260 270 280  DGPVLLPDNH YLSTQSALSK DPNEKRDHMV LLEFVTAAGI TLGMDELYKS GIR**GGSGGS**X XXXXXXXXXX  290 300 310 320 330 340 350  X**GGSGGS**TMV SKGEELFTGV VPILVELDGD VNGHKFSVSG EGEGDATYGK LTLKFICTTG KLPVPWPTLV  360 370 380 390 400 410 420  TTFGYGLQCF ARYPDHMKQH DFFKSAMPEG YVQERTIFFK DDGNYKTRAE VKFEGDTLVN RIELKGIDFK  430 440 450 460 470 480 490  EDGNILGHKL EYNYNSHNVY IMADKQKNGI KVNFKIRHNI EDGSVQLADH YQQNTPIGDG PVLLPDNHYL  500 510 520 525  SYQSALSKDP NEKRDHMVLL EFVTAAGITL GMDELYK |
| Codon optimized CLY2 gene sequence including N-terminal His_6_-Th-tag  His_6_-tag  ECFP  **GGSGGS rpt**  Mpro cut site (XXX)  EYFP | 5’‑ ATGGGCAGCAGCCATCATCATCATCATCACAGCAGCGGCCTGGTGCCGCGCGGCAGCCATATGGTGAGCAAGGGCGAGGAGCTGTTCACCGGGGTGGTGCCCATCCTGGTCGAGCTGGACGGCGACGTAAACGGCCACAAGTTCAGCGTGTCCGGCGAGGGCGAGGGCGATGCCACCTACGGCAAGCTGACCCTGAAGTTCATCTGCACCACCGGCAAGCTGCCCGTGCCCTGGCCCACCCTCGTGACCACCCTGACCTGGGGCGTGCAGTGCTTCAGCCGCTACCCCGACCACATGAAGCAGCACGACTTCTTCAAGTCCGCCATGCCCGAAGGCTACGTCCAGGAGCGCACCATCTTCTTCAAGGACGACGGCAACTACAAGACCCGCGCCGAGGTGAAGTTCGAGGGCGACACCCTGGTGAACCGCATCGAGCTGAAGGGCATCGACTTCAAGGAGGACGGCAACATCCTGGGGCACAAGCTGGAGTACAACTACATCAGCCACAACGTCTATATCACCGCCGACAAGCAGAAGAACGGCATCAAGGCCAACTTCAAGATCCGCCACAACATCGAGGACGGCAGCGTGCAGCTCGCCGACCACTACCAGCAGAACACCCCCATCGGCGACGGCCCCGTGCTGCTGCCCGACAACCACTACCTGAGCACCCAGTCCGCCCTGAGCAAAGACCCCAACGAGAAGCGCGATCACATGGTCCTGCTGGAGTTCGTGACCGCCGCCGGGATCACTCTCGGCATGGACGAGCTGTACAAGTCCGGAATTCGT**GGTGGATCCGGTGGTAGT**XXXXXXXXXXXXXXXXXXXXXXXXXXXXXXXXXXXX**GGTGGATCCGGAGGTAGC**ACCATGGTGAGCAAGGGCGAGGAGCTGTTCACCGGGGTGGTGCCCATCCTGGTCGAGCTGGACGGCGACGTAAACGGCCACAAGTTCAGCGTGTCCGGCGAGGGCGAGGGCGATGCCACCTACGGCAAGCTGACCCTGAAGTTCATCTGCACCACCGGCAAGCTGCCCGTGCCCTGGCCCACCCTCGTGACCACCTTCGGCTACGGCCTGCAGTGCTTCGCCCGCTACCCCGACCACATGAAGCAGCACGACTTCTTCAAGTCCGCCATGCCCGAAGGCTACGTCCAGGAGCGCACCATCTTCTTCAAGGACGACGGCAACTACAAGACCCGCGCCGAGGTGAAGTTCGAGGGCGACACCCTGGTGAACCGCATCGAGCTGAAGGGCATCGACTTCAAGGAGGACGGCAACATCCTGGGGCACAAGCTGGAGTACAACTACAACAGCCACAACGTCTATATCATGGCCGACAAGCAGAAGAACGGCATCAAGGTGAACTTCAAGATCCGCCACAACATCGAGGACGGCAGCGTGCAGCTCGCCGACCACTACCAGCAGAACACCCCCATCGGCGACGGCCCCGTGCTGCTGCCCGACAACCACTACCTGAGCTACCAGTCCGCCCTGAGCAAAGACCCCAACGAGAAGCGCGATCACATGGTCCTGCTGGAGTTCGTGACCGCCGCCGGGATCACTCTCGGCATGGACGAGCTGTACAAGTAA -3’ |
|  |  |
| pET28-ECFP plasmid expresses His6-Th-ECFP-(GGSGGS) fusion protein  His_6_-tag  Thrombin Cut Site (￬)  ECFP  **GGSGGS rpt** | 10 ￬20 30 40 50 60 70  **MGSSHHHHHH** **SSGLVPRGSH** MVSKGEELFT GVVPILVELD GDVNGHKFSV SGEGEGDATY GKLTLKFICT  80 90 100 110 120 130 140  TGKLPVPWPT LVTTLTWGVQ CFSRYPDHMK QHDFFKSAMP EGYVQERTIF FKDDGNYKTR AEVKFEGDTL  150 160 170 180 190 200 210  VNRIELKGID FKEDGNILGH KLEYNYISHN VYITADKQKN GIKANFKIRH NIEDGSVQLA DHYQQNTPIG  220 230 240 250 260 269  DGPVLLPDNH YLSTQSALSK DPNEKRDHMV LLEFVTAAGI TLGMDELYKS GIR**GGSGGS** |
| Codon optimized ECFP gene sequence including N-terminal Th-His_6-_tag and C-terminal GGSGGS linker  His_6_-tag  ECFP  **GGSGGS rpt** | 5’‑ ATGGGCAGCAGCCATCATCATCATCATCACAGCAGCGGCCTGGTGCCGCGCGGCAGCCATATGGTGAGCAAGGGCGAGGAGCTGTTCACCGGGGTGGTGCCCATCCTGGTCGAGCTGGACGGCGACGTAAACGGCCACAAGTTCAGCGTGTCCGGCGAGGGCGAGGGCGATGCCACCTACGGCAAGCTGACCCTGAAGTTCATCTGCACCACCGGCAAGCTGCCCGTGCCCTGGCCCACCCTCGTGACCACCCTGACCTGGGGCGTGCAGTGCTTCAGCCGCTACCCCGACCACATGAAGCAGCACGACTTCTTCAAGTCCGCCATGCCCGAAGGCTACGTCCAGGAGCGCACCATCTTCTTCAAGGACGACGGCAACTACAAGACCCGCGCCGAGGTGAAGTTCGAGGGCGACACCCTGGTGAACCGCATCGAGCTGAAGGGCATCGACTTCAAGGAGGACGGCAACATCCTGGGGCACAAGCTGGAGTACAACTACATCAGCCACAACGTCTATATCACCGCCGACAAGCAGAAGAACGGCATCAAGGCCAACTTCAAGATCCGCCACAACATCGAGGACGGCAGCGTGCAGCTCGCCGACCACTACCAGCAGAACACCCCCATCGGCGACGGCCCCGTGCTGCTGCCCGACAACCACTACCTGAGCACCCAGTCCGCCCTGAGCAAAGACCCCAACGAGAAGCGCGATCACATGGTCCTGCTGGAGTTCGTGACCGCCGCCGGGATCACTCTCGGCATGGACGAGCTGTACAAGTCCGGAATTCGT**GGTGGATCCGGTGGTAGTTAA** -3’ |
|  |  |
| pET28-EYFP plasmid expresses (GGSGGS)-EYFP-Th-His fusion protein  **GGSGGS rpt**  EYFP  Thrombin Cut Site (￬)  His_6_-tag | 10 20 30 40 50 60 70 M**GGSGGS**TMV SKGEELFTGV VPILVELDGD VNGHKFSVSG EGEGDATYGK LTLKFICTTG KLPVPWPTLV   80 90 100 110 120 130 140 TTFGYGLQCF ARYPDHMKQH DFFKSAMPEG YVQERTIFFK DDGNYKTRAE VKFEGDTLVN RIELKGIDFK   150 160 170 180 190 200 210 EDGNILGHKL EYNYNSHNVY IMADKQKNGI KVNFKIRHNI EDGSVQLADH YQQNTPIGDG PVLLPDNHYL   220 230 240 250 ￬ 260 261 SYQSALSKDP NEKRDHMVLL EFVTAAGITL GMDELYKLVP RGSLEHHHHH H |
| Codon optimized EYFP gene sequence including N-terminal GGSGGS linker and C-terminal Th-His_6-_tag  **GGSGGS rpt**  EYFP  His_6_-tag | 5’‑ ATG**GGTGGATCCGGAGGTAGC**ACCATGGTGAGCAAGGGCGAGGAGCTGTTCACCGGGGTGGTGCCCATCCTGGTCGAGCTGGACGGCGACGTAAACGGCCACAAGTTCAGCGTGTCCGGCGAGGGCGAGGGCGATGCCACCTACGGCAAGCTGACCCTGAAGTTCATCTGCACCACCGGCAAGCTGCCCGTGCCCTGGCCCACCCTCGTGACCACCTTCGGCTACGGCCTGCAGTGCTTCGCCCGCTACCCCGACCACATGAAGCAGCACGACTTCTTCAAGTCCGCCATGCCCGAAGGCTACGTCCAGGAGCGCACCATCTTCTTCAAGGACGACGGCAACTACAAGACCCGCGCCGAGGTGAAGTTCGAGGGCGACACCCTGGTGAACCGCATCGAGCTGAAGGGCATCGACTTCAAGGAGGACGGCAACATCCTGGGGCACAAGCTGGAGTACAACTACAACAGCCACAACGTCTATATCATGGCCGACAAGCAGAAGAACGGCATCAAGGTGAACTTCAAGATCCGCCACAACATCGAGGACGGCAGCGTGCAGCTCGCCGACCACTACCAGCAGAACACCCCCATCGGCGACGGCCCCGTGCTGCTGCCCGACAACCACTACCTGAGCTACCAGTCCGCCCTGAGCAAAGACCCCAACGAGAAGCGCGATCACATGGTCCTGCTGGAGTTCGTGACCGCCGCCGGGATCACTCTCGGCATGGACGAGCTGTACAAGCTGGTGCCGCGCGGCAGCCTCGAGCACCACCACCACCACCACTGA-3’ |
| C4 – TSAVLQSGFRKM |  |
| fwd | 5’‑ GGTGAACAGCTCCTCGCCCTTGCTCACCATGGTGCTACCTCCGGATCCACCCATTTTGCG -3’ |
| insert | 5’‑ TCCGGTGGTAGTACCAGCGCGGTGCTGCAGAGCGGCTTTCGCAAAATGGGTGGATCCGGA -3’ |
| rev | 5’‑ CGGCATGGACGAGCTGTACAAGTCCGGAATTCGTGGTGGATCCGGTGGTAGTACCAGCGC -3’ |
| assembled | 5’‑ CGGCATGGACGAGCTGTACAAGTCCGGAATTCGT**GGTGGATCCGGTGGTAGT**ACCAGCGCGGTGCTGCAGAGCGGCTTTCGCAAAATG**GGTGGATCCGGAGGTAGC**ACCATGGTGAGCAAGGGCGAGGAGCTGTTCACC -3’ |
| C5 – SGVTFQSAVKRT |  |
| fwd | 5’‑ CGGTGAACAGCTCCTCGCCCTTGCTCACCATGGTGCTACCTCCGGATCCACCGGTGC -3’ |
| insert | 5’‑ GGTGGTAGTAGCGGCGTGACCTTTCAGAGCGCGGTGAAACGCACCGGTGGATCCGGA -3’ |
| rev | 5’‑ CATGGACGAGCTGTACAAGTCCGGAATTCGTGGTGGATCCGGTGGTAGTAGCGGCGTGAC -3’ |
| assembled | 5’‑ CATGGACGAGCTGTACAAGTCCGGAATTCGT**GGTGGATCCGGTGGTAGT**AGCGGCGTGACCTTTCAGAGCGCGGTGAAACGCACC**GGTGGATCCGGAGGTAGC**ACCATGGTGAGCAAGGGCGAGGAGCTGTTCACCG -3’ |
| C6 – KVATVQSKMSDV |  |
| fwd | 5’‑ CGGTGAACAGCTCCTCGCCCTTGCTCACCATGGTGCTACCTCCGGATCCACCCACATCGC -3’ |
| insert | 5’‑ TCCGGTGGTAGTAAAGTGGCGACCGTGCAGAGCAAAATGAGCGATGTGGGTGGATCCGGA -3’ |
| rev | 5’‑ CATGGACGAGCTGTACAAGTCCGGAATTCGTGGTGGATCCGGTGGTAGTAAAGTGGCGAC -3’ |
| assembled | 5’‑ CATGGACGAGCTGTACAAGTCCGGAATTCGT**GGTGGATCCGGTGGTAGT**AAAGTGGCGACCGTGCAGAGCAAAATGAGCGATGTG**GGTGGATCCGGAGGTAGC**ACCATGGTGAGCAAGGGCGAGGAGCTGTTCACCG -3’ |
| C7 – NRATLQAIASEF |  |
| fwd | 5’‑ CGGTGAACAGCTCCTCGCCCTTGCTCACCATGGTGCTACCTCCGGATCCACCAAATTCGC -3’ |
| insert | 5’‑ GGTGGTAGTAACCGCGCGACCCTGCAGGCGATTGCGAGCGAATTTGGTGGATCCGGAGGT -3’ |
| rev | 5’‑ GGACGAGCTGTACAAGTCCGGAATTCGTGGTGGATCCGGTGGTAGTAACCGCGCGACC -3’ |
| assembled | 5’‑ GGACGAGCTGTACAAGTCCGGAATTCGT**GGTGGATCCGGTGGTAGT**AACCGCGCGACCCTGCAGGCGATTGCGAGCGAATTT**GGTGGATCCGGAGGTAGC**ACCATGGTGAGCAAGGGCGAGGAGCTGTTCACCG -3’ |
| C8 – SAVKLQNNELSP |  |
| fwd | 5’‑ GGTGAACAGCTCCTCGCCCTTGCTCACCATGGTGCTACCTCCGGATCCACCCGGGC -3’ |
| insert | 5’‑ TCCGGTGGTAGTAGCGCGGTGAAACTGCAGAACAACGAACTGAGCCCGGGTGGATCCGGA -3’ |
| rev | 5’‑ CGGCATGGACGAGCTGTACAAGTCCGGAATTCGTGGTGGATCCGGTGGTAGTAGCGCGG -3’ |
| assembled | 5’‑ CGGCATGGACGAGCTGTACAAGTCCGGAATTCGT**GGTGGATCCGGTGGTAGT**AGCGCGGTGAAACTGCAGAACAACGAACTGAGCCCG**GGTGGATCCGGAGGTAGC**ACCATGGTGAGCAAGGGCGAGGAGCTGTTCACC -3’ |
| C9 – ATVRLQAGNATE |  |
| fwd | 5’‑ GGTGAACAGCTCCTCGCCCTTGCTCACCATGGTGCTACCTCCGGATCCACCCATTTTGCG -3’ |
| insert | 5’‑ TCCGGTGGTAGTGCGACCGTGCGCCTGCAGGCGGGCAACGCGACCGAAGGTGGATCCGGA -3’ |
| rev | 5’‑ CGGCATGGACGAGCTGTACAAGTCCGGAATTCGTGGTGGATCCGGTGGTAGTGCGACCG -3’ |
| assembled | 5’‑ CGGCATGGACGAGCTGTACAAGTCCGGAATTCGT**GGTGGATCCGGTGGTAGT**GCGACCGTGCGCCTGCAGGCGGGCAACGCGACCGAA**GGTGGATCCGGAGGTAGC**ACCATGGTGAGCAAGGGCGAGGAGCTGTTCACC -3’ |
| C10 – REPMLQSADAQS |  |
| fwd | 5’‑ CGGTGAACAGCTCCTCGCCCTTGCTCACCATGGTGCTACCTCCGGATCCACCGCTCTGC -3’ |
| insert | 5’‑ TCCGGTGGTAGTCGCGAACCGATGCTGCAGAGCGCGGATGCGCAGAGCGGTGGATCCGGA -3’ |
| rev | 5’‑ CATGGACGAGCTGTACAAGTCCGGAATTCGTGGTGGATCCGGTGGTAGTCGCGAACC -3’ |
| assembled | 5’‑ CATGGACGAGCTGTACAAGTCCGGAATTCGT**GGTGGATCCGGTGGTAGT**CGCGAACCGATGCTGCAGAGCGCGGATGCGCAGAGC**GGTGGATCCGGAGGTAGC**ACCATGGTGAGCAAGGGCGAGGAGCTGTTCACCG -3’ |
| C12 – PHTVLQAVGACV |  |
| fwd | 5’‑ CGGTGAACAGCTCCTCGCCCTTGCTCACCATGGTGCTAGATCCACCCACGCACGCG -3’ |
| insert | 5’‑ TCCGGTGGTAGTCCGCATACCGTGCTGCAGGCGGTGGGCGCGTGCGTGGGTGGATC -3’ |
| rev | 5’‑ GGCATGGACGAGCTGTACAAGTCCGGAATTCGTGGTGGATCCGGTGGTAGTCCGCATACC -3’ |
| assembled | 5’‑ GGCATGGACGAGCTGTACAAGTCCGGAATTCGT**GGTGGATCCGGTGGTAGT**CCGCATACCGTGCTGCAGGCGGTGGGCGCGTGCGTG**GGTGGATCCGGAGGTAGC**ACCATGGTGAGCAAGGGCGAGGAGCTGTTCACCG -3’ |
| C13 – NVATLQAENVTG |  |
| fwd | 5’‑ CGGTGAACAGCTCCTCGCCCTTGCTCACCATGGTGCTACCTCCGGATCCACCGCCGG -3’ |
| insert | 5’‑ TCCGGTGGTAGTAACGTGGCGACCCTGCAGGCGGAAAACGTGACCGGCGGTGGATCCGGA -3’ |
| rev | 5’‑ GGCATGGACGAGCTGTACAAGTCCGGAATTCGTGGTGGATCCGGTGGTAGTAACGTGGCG -3’ |
| assembled | 5’‑ GGCATGGACGAGCTGTACAAGTCCGGAATTCGT**GGTGGATCCGGTGGTAGT**AACGTGGCGACCCTGCAGGCGGAAAACGTGACCGGC**GGTGGATCCGGAGGTAGC**ACCATGGTGAGCAAGGGCGAGGAGCTGTTCACCG -3’ |
| C14 – TFTRLQSLENVA |  |
| fwd | 5’‑ CAGCTCCTCGCCCTTGCTCACCATGGTGCTACCTCCGGATCCACCCGCCACGTTTTCCAG -3’ |
| insert | 5’‑ TCCGGTGGTAGTACCTTTACCCGCCTGCAGAGCCTGGAAAACGTGGCGGGTGGA -3’ |
| rev | 5’‑ GGACGAGCTGTACAAGTCCGGAATTCGTGGTGGATCCGGTGGTAGTACCTTTACCCGC -3’ |
| assembled | 5’‑ GGCATGGACGAGCTGTACAAGTCCGGAATTCGT**GGTGGATCCGGTGGTAGT**ACCTTTACCCGCCTGCAGAGCCTGGAAAACGTGGCG**GGTGGATCCGGAGGTAGC**ACCATGGTGAGCAAGGGCGAGGAGCTG -3’ |
| C15 – FYPKLQSSQAWQ |  |
| fwd | 5’‑ CGGTGAACAGCTCCTCGCCCTTGCTCACCATGGTGCTACCTCCGGATCCACCCTGCCACG -3’ |
| insert | 5’‑ TCCGGTGGTAGTTTTTATCCGAAACTGCAGAGCAGCCAGGCGTGGCAGGGTGGATCCGGA -3’ |
| rev | 5’‑ GAGCTGTACAAGTCCGGAATTCGTGGTGGATCCGGTGGTAGTTTTTATCCGAAACTGCAG -3’ |
| assembled | 5’‑ GAGCTGTACAAGTCCGGAATTCGT**GGTGGATCCGGTGGTAGT**TTTTATCCGAAACTGCAGAGCAGCCAGGCGTGGCAG**GGTGGATCCGGAGGTAGC**ACCATGGTGAGCAAGGGCGAGGAGCTGTTCACCG -3’ |
|  |  |

| **Table S4. Polyprotein P6-P1 cut site sequence binding in published structures** | | | | | |
| --- | --- | --- | --- | --- | --- |
| **Cut Site**  (PBD ID) | Number of residues present in model | Accessible Surface Area**^‡^** (Å^2^) ^4^ | Buried Surface Area**^‡^** (Å^2^) ^4^ | Average B-factor**^‡^**(Å^2^) | Hydrophobicity^3^ |
| **C4** |  | **867** | **601** | **36** | **0.80** |
| 7MB4^2^ | 6 | 909 | 626 | 30 |  |
| 7T70^2^ | 6 | 846 | 578 | 27 |  |
| 8DRR^21^ | 6 | 861 | 624 | 55 |  |
| 7DVP^22^ | 6 | 852 | 575 | 32 |  |
| **C5** |  | **857** | **571** | **33** | **0.27** |
| 7JOY^23^ | 6 | 818 | 452 | 39 |  |
| 7MB5^2^ | 6 | 918 | 647 | 26 |  |
| 7T8M^2^ | 5 | 731 | 561 | 17 |  |
| 7DVW^22^ | 6 | 816 | 529 | 50 |  |
| **C6** |  | **923** | **605** | **51** | **0.35** |
| 7MB6^2^ | 6 | 926 | 610 | 58 |  |
| 8DRT^21^ | 6 | 920 | 601 | 58 |  |
| 7DVX^22^ | 6 | 868 | 555 | 35 |  |
| **C7** |  | **989** | **633** | **46** | **-1.10** |
| 7MB7^2^ | 6 | 1014 | 672 | 54 |  |
| 7T8R^2^ | 6 | 974 | 595 | 32 |  |
| 8DRU^21^ | 6 | 979 | 632 | 52 |  |
| **C8** |  | **898** | **626** | **35** | **0.27** |
| 7MB8^2^ | 6 | 948 | 652 | 26 |  |
| 7T9Y^2^ | 6 | 854 | 583 | 30 |  |
| 8DRV^21^ | 6 | 890 | 642 | 48 |  |
| **C9** |  | **855** | **626** | **39** | **0.18** |
| 7TA4^2^ | 6 | 843 | 590 | 29 |  |
| 8DRW^21^ | 6 | 866 | 662 | 54 |  |
| 7DVY^22^ | 6 | 911 | 618 | 34 |  |
| **C10** |  | **979** | **621** | 37 | **-1.23** |
| 7MB9^2^ | 6 | 1077 | 676 | 39 |  |
| 7TA7^2^ | 6 | 881 | 566 | 35 |  |
| **C12** |  | **896** | **618** | **37** | **-0.17** |
| 7TB2^2^ | 6 | 903 | 599 | 28 |  |
| 8DRY^21^ | 6 | 888 | 637 | 45 |  |
| **C13** |  | **877** | **599** | **38** | **0.35** |
| 7TBT^2^ | 6 | 893 | 573 | 40 |  |
| 8DRZ^21^ | 6 | 861 | 625 | 35 |  |
| **C14** |  | **981** | **648** | **69** | **-0.47** |
| 7DW0^22^ | 6 | 981 | 648 | 69 |  |
| **C15** |  | **1060** | **660** | **35** | **-0.62** |
| 7TC4^2^ | 6 | 1065 | 615 | 26 |  |
| 7DW6^22^ | 5 | 879 | 596 | 43 |  |
| **^‡^**Bolded values show average values across all analyzed models for each cut site sequence. | | | | | |

**Supplementary References**

1. Pérez-Vargas, J. *et al.* A novel class of broad-spectrum active-site-directed 3C-like protease inhibitors with nanomolar antiviral activity against highly immune-evasive SARS-CoV-2 Omicron subvariants. *Emerg. Microbes Infect.* **0**, 2246594 (2023).

2. Shaqra, A. M. *et al.* Defining the substrate envelope of SARS-CoV-2 main protease to predict and avoid drug resistance. *Nat. Commun.* **13**, 3556 (2022).

3. Kyte, J. & Doolittle, R. F. A simple method for displaying the hydropathic character of a protein. *J. Mol. Biol.* **157**, 105–132 (1982).

4. Krissinel, E. & Henrick, K. Inference of macromolecular assemblies from crystalline state. *J. Mol. Biol.* **372**, 774–797 (2007).

5. Legare, S., Heide, F., Bailey-Elkin, B. A. & Stetefeld, J. Improved SARS-CoV-2 main protease high-throughput screening assay using a 5-carboxyfluorescein substrate. *J. Biol. Chem.* **298**, 101739 (2022).

6. Dražić, T., Kühl, N., Leuthold, M. M., Behnam, M. A. M. & Klein, C. D. Efficiency Improvements and Discovery of New Substrates for a SARS-CoV-2 Main Protease FRET Assay. *SLAS Discov. Adv. Life Sci. R D* **26**, 1189–1199 (2021).

7. Breidenbach, J. *et al.* Targeting the Main Protease of SARS-CoV-2: From the Establishment of High Throughput Screening to the Design of Tailored Inhibitors. *Angew. Chem. Int. Ed.* **60**, 10423–10429 (2021).

8. Rut, W. *et al.* SARS-CoV-2 Mpro inhibitors and activity-based probes for patient-sample imaging. *Nat. Chem. Biol.* **17**, 222–228 (2021).

9. MacDonald, E. A. *et al.* Recognition of Divergent Viral Substrates by the SARS-CoV-2 Main Protease. *ACS Infect. Dis.* **7**, 2591–2595 (2021).

10. Zhu, W. *et al.* Identification of SARS-CoV-2 3CL Protease Inhibitors by a Quantitative High-Throughput Screening. *ACS Pharmacol. Transl. Sci.* **3**, 1008–1016 (2020).

11. Sacco, M. D. *et al.* Structure and inhibition of the SARS-CoV-2 main protease reveal strategy for developing dual inhibitors against Mpro and cathepsin L. *Sci. Adv.* **6**, eabe0751 (2020).

12. Ullrich, S. *et al.* Challenges of short substrate analogues as SARS-CoV-2 main protease inhibitors. *Bioorg. Med. Chem. Lett.* **50**, 128333 (2021).

13. Ma, C. *et al.* Boceprevir, GC-376, and calpain inhibitors II, XII inhibit SARS-CoV-2 viral replication by targeting the viral main protease. *Cell Res.* **30**, 678–692 (2020).

14. Kuo, C.-J. *et al.* Kinetic Characterization and Inhibitor Screening for the Proteases Leading to Identification of Drugs against SARS-CoV-2. *Antimicrob. Agents Chemother.* **65**, 10.1128/aac.02577-20 (2021).

15. Dai, W. *et al.* Structure-based design of antiviral drug candidates targeting the SARS-CoV-2 main protease. *Science* **368**, 1331–1335 (2020).

16. Jin, Z. *et al.* Structure of M pro from SARS-CoV-2 and discovery of its inhibitors. *Nature* **582**, 289–293 (2020).

17. Li, Z. *et al.* Identify potent SARS-CoV-2 main protease inhibitors via accelerated free energy perturbation-based virtual screening of existing drugs. *Proc. Natl. Acad. Sci. U. S. A.* **117**, 27381 (2020).

18. Costanzi, E. *et al.* Structural and Biochemical Analysis of the Dual Inhibition of MG-132 against SARS-CoV-2 Main Protease (Mpro/3CLpro) and Human Cathepsin-L. *Int. J. Mol. Sci.* **22**, 11779 (2021).

19. Liu, C. *et al.* Dual inhibition of SARS-CoV-2 and human rhinovirus with protease inhibitors in clinical development. *Antiviral Res.* **187**, 105020 (2021).

20. Krichel, B., Falke, S., Hilgenfeld, R., Redecke, L. & Uetrecht, C. Processing of the SARS-CoV pp1a/ab nsp7-10 region. *Biochem. J.* **477**, 1009–1019 (2020).

21. Lee, J. *et al.* X-ray crystallographic characterization of the SARS-CoV-2 main protease polyprotein cleavage sites essential for viral processing and maturation. *Nat. Commun.* **13**, 5196 (2022).

22. Zhao, Y. *et al.* Structural basis for replicase polyprotein cleavage and substrate specificity of main protease from SARS-CoV-2. *Proc. Natl. Acad. Sci.* **119**, e2117142119 (2022).

23. Lee, J. *et al.* Crystallographic structure of wild-type SARS-CoV-2 main protease acyl-enzyme intermediate with physiological C-terminal autoprocessing site. *Nat. Commun.* **11**, 5877 (2020).
